# Development of hRad51–Cas9 nickase fusions that mediate HDR without double-stranded breaks

**DOI:** 10.1038/s41467-019-09983-4

**Published:** 2019-05-17

**Authors:** Holly A. Rees, Wei-Hsi Yeh, David R. Liu

**Affiliations:** 1 Merkin Institute of Transformative Technologies in Healthcare, Broad Institute of Harvard and MIT, Cambridge, MA 02142 USA; 2 Howard Hughes Medical Institute, Harvard University, Cambridge, MA 02142 USA; 3 Department of Chemistry and Chemical Biology, Harvard University, Cambridge, MA 02138 USA; 4 Program in Speech and Hearing Bioscience and Technology, Harvard Medical School, Boston, MA 02115 USA

**Keywords:** Genetic engineering, Gene targeting

## Abstract

In mammalian cells, double-stranded DNA breaks (DSBs) are preferentially repaired through end-joining processes that generally lead to mixtures of insertions and deletions (indels) or other rearrangements at the cleavage site. In the presence of homologous DNA, homology-directed repair (HDR) can generate specific mutations, albeit typically with modest efficiency and a low ratio of HDR products:indels. Here, we develop hRad51 mutants fused to Cas9(D10A) nickase (RDN) that mediate HDR while minimizing indels. We use RDN to install disease-associated point mutations in HEK293T cells with comparable or better efficiency than Cas9 nuclease and a 2.7-to-53-fold higher ratio of desired HDR product:undesired byproducts. Across five different human cell types, RDN variants generally result in higher HDR:indel ratios and lower off-target activity than Cas9 nuclease, although HDR efficiencies remain strongly site- and cell type-dependent. RDN variants provide precision editing options in cell types amenable to HDR, especially when byproducts of DSBs must be minimized.

## Abstract (web summary)

Here the authors fuse hRad51 and variants thereof to Cas9 nickase to facilitate homology-directed repair without generating double strand breaks, minimizing indel formation and off-target editing. This tool represents progress towards the goal of performing HDR without an excess of undesired side products.

## Introduction

**Fig. 1:**
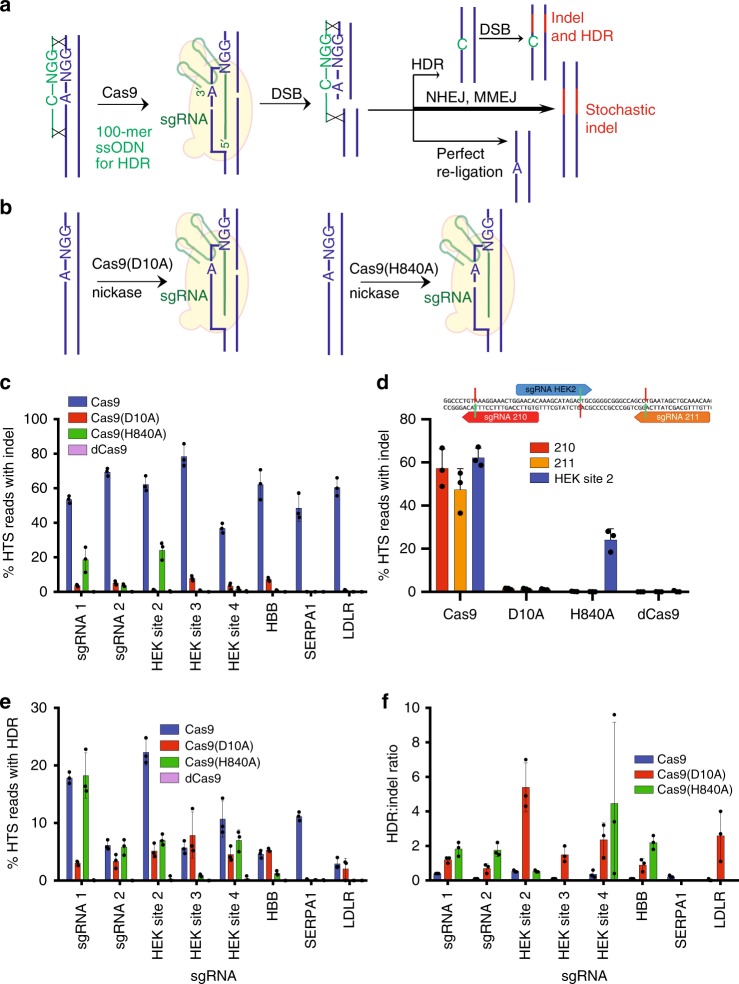
Indel formation and HDR in HEK293T cells mediated by Cas9 or Cas9 nickases. **a** DSB-mediated HDR using Cas9 and a 100-mer ssODN. **b** DNA nicks resulting from Cas9(D10A) or Cas9(H840A) nickase. **c** Indels resulting from Cas9 nuclease, Cas9 nickase, or dead Cas9 at eight loci in HEK293T cells. **d** Comparison of indel frequencies associated with three sgRNAs in close proximity. The sgRNA sequences used in are shown, with red arrows indicating nicks induced by Cas9(D10A) nickase, and green arrows showing nicks by Cas9(H840A) nickase. **e** HDR frequencies, measured by high-throughput DNA sequencing of unsorted HEK293T cells at eight endogenous genomic loci. **f** HDR:indel ratio associated with editing at eight loci. All data are shown as individual data points and mean ± s.d. for *n* = 3 independent biological replicates, performed on different days. Source data are provided in the Source Data file

Widely used genome editing strategies include gene disruption by generating insertions and deletions (indels) at a targeted locus following a double-stranded DNA break (DSB)^1^, homology-directed repair (HDR) following a targeted DSB^2^, and base editing, which enables the precise installation of transition point mutations (C to T, G to A, A to G, or T to C) without creating DSBs^3–5^. Among these three strategies, HDR offers access to the broadest possible range of changes to genomic DNA in mammalian cells (Fig. 1a)^6^. The use of single-stranded DNA oligonucleotides containing PAM-blocking mutations as donor templates can improve HDR outcomes by preventing re-cutting of the target site after successful HDR (Fig. 1a)^7^. Nevertheless, because HDR is usually initiated by a DSB, HDR is accompanied by undesired cellular side-effects including high levels of indel formation^7,8^, DNA translocations^9^, large deletions^10^, and p53 activation^11,12^.

We sought to improve ratios of desired:undesired HDR products by exploring the initiation of HDR from a DNA nick rather than a DSB. In contrast to DSBs, DNA nicks generally do not induce undesired genome modification^13–15^, a principle exploited by base editors to minimize editing byproducts^3,5,16^. Mutating catalytic residues in programmable nucleases can result in programmable nickases that cleave only one of the two strands of DNA at the target locus^17–20^. Although single nicks can lead to more favorable HDR:indel ratios than double-stranded DNA breaks^19,21,22^, nicks usually lead to much lower frequencies of genome editing when compared to DSBs (typically 5–20-fold)^23^, making nickases substantially less useful than nucleases as genome editing tools^17,20,22,24^.

In this study we achieve DSB-free HDR with minimal byproducts and reduced off-target editing by fusing hRad51 variants to a programmable nickase to generate hRad51–Cas9 (D10A) nickase fusions (RDN variants). We chose hRad51 due to its known involvement in the repair of nicked DNA^17,22^. RDN is capable of stimulating HDR at a DNA nick, resulting in a much higher ratio of HDR product:indel formation in human cells (up to 53-fold at the eight genomic loci tested here), substantially lower off-target editing. A known mutant of hRad51 that cannot bind BRCA2^25,26^ can be used in RDN to further increase the HDR:indel ratio. A second known hRad51 mutant that cannot self-associate^25,26^ increases overall HDR efficiency while slightly lowering HDR:indel ratios. RDN-mediated HDR is a one-step procedure that does not require inclusion of PAM-blocking mutations^7^ and can use readily synthesized 100-mer single stranded DNA (ssDNA) oligonucleotides as donor templates. Although RDN remains limited by its dependence on cellular DNA repair processes underlying HDR, RDN may be useful for applications that require precise genome edits not accessible to base editing while minimizing undesired consequences of DSBs.

## Results

### Indels caused by single Cas9 nickases

Cas9 contains two independent nuclease domains, either of which can be disabled to generate a nickase that selectively cleaves either the guide RNA-paired strand (Cas9(D10A) nickase) or the opposite strand (Cas9(H840A) nickase) (Fig. 1b)^27^. We used high-throughput DNA sequencing (HTS) to systematically compare the editing outcomes of Cas9, Cas9(D10A), or Cas9(H840A) nickases at eight genomic loci in three human cell lines.

While both nickases resulted in substantially fewer indels than intact Cas9, nick-induced indel formation was highly strand-dependent and locus-dependent (Fig. 1c). The Cas9(D10A) and Cas9(H840A) nickases displayed different relative activities when paired with different sgRNAs; for example, at HEK site 2 the Cas9(H840A) nickase generated 24 ± 5% indels (± values represent standard deviations for three biological replicates) and the Cas9(D10A) nickase generated only 1.1 ± 0.2% indels, while at HEK site 3 Cas9(H840A) nickase resulted in only 0.73 ± 0.38% indels but Cas9(D10A) nickase treatment generated 7.9 ± 1.4% indels (Fig. 1c). One of the eight sgRNAs we tested, targeted to the SERPA1 locus, did not lead to detectable indels when combined with either nickase despite robust indel formation when combined with Cas9 (Fig. 1c). A similar pattern of indel formation at nicked sites was observed in HeLa and U2OS cells (Supplementary Fig. 1a, b), and with the ABEmax base editor, which contains a Cas9(D10A) nickase, although other base editors resulted in reduced indel frequencies compared to their component nickase domains alone (Supplementary Fig. 6 and Supplementary Note 1). Observed indel frequencies did not correlate with the presence of microhomology as predicted using inDelphi^28^ (Supplementary Fig. 2b). These results suggest that the cellular response to single nick generation is site-dependent and unpredictable by microhomology, though in general leads to substantially lower indel formation than the cellular response to DSBs.

We hypothesized that the site dependence of nickase-induced indels could be explained if the induced nicks were converted to DSBs by a separate cellular process such as DNA replication. When a replication fork encounters a nick, it becomes a DSB^29^. To test this possibility, we analyzed two sgRNAs (211 and 210) that target DNA either 28 bp upstream (sgRNA 210) or 18 bp downstream (sgRNA 211) of HEK site 2, a particularly asymmetric locus that results in high levels of Cas9(H840A) nickase-mediated indels but low levels of Cas9(D10A) nickase-induced indels. While Cas9(H840A) nickase and the HEK site 2 sgRNA resulted in high indel levels (24 ± 5%), nicking the same strand slightly upstream or downstream of HEK site 2 resulted in 17-fold lower indel formation (Fig. 1d). These observations indicate that the high indel frequency generated by Cas9(H840A) nickase when paired with the HEK site 2 sgRNA is strongly dependent on the exact site being targeted. These data suggest that the cellular response to nicks is highly sgRNA-dependent. The high degree of sgRNA-dependence associated with nick-induced indels may explain previously conflicting reports of the relative inactivity of the H840A nickase in human cells^30–32^.

### HDR stimulated by single Cas9 nickases

The use of HDR for precision genome editing in mammalian cells is limited by low efficiency in many cell types (T cells being a notable exception^33^), and the excess of indels and other undesired cellular outcomes that result from DSB formation. Previous work with Cas9 nickases^18,20,22,24,32^, homing endonucleases converted to nickases^17^, and zinc finger nickases^19^ demonstrates that nicks can induce low levels of HDR when combined with a donor DNA template.

We wondered whether the observed variability among nick-induced indel formation also applies to nick-induced HDR. To assess this possibility, we designed 100-mer single-stranded DNA oligonucleotide (ssODN) templates for each of eight genomic loci and co-delivered them with Cas9 nuclease, Cas9 nickases, and catalytically dead Cas9 (dCas9). For three loci (*HBB*, *SERPA1*, and *LDLR*), the ssODN encoded a single human pathogenic SNP located in the protospacer. For the remaining five loci, the donor templates were designed to incorporate an SNP within the protospacer as well as a PAM-altering SNP, as described in the CORRECT method for HDR donor template design^7^. We lipofected a plasmid encoding Cas9, Cas9 nickase, or dead Cas9, a plasmid expressing the indicated sgRNA, and the corresponding ssODN donor template into HEK293T cells. Four days post-lipofection, genomic DNA was purified and analyzed by high throughput sequencing (HTS). We used Crispresso2^34,35^ to filter out reads containing indels from our alignment prior to assessing HDR efficiency to ensure that reads containing both indels and HDR did not contribute to tabulated HDR efficiencies. DNA strands that contained both indels and HDR events were counted as indels during tabulations (see Methods section).

At seven of eight sites, we detected HDR with one or both Cas9 nickases (Fig. 1e). Linear regression analysis identified a weak positive correlation (*R*^2^ = 0.57, *p* = 0.031 for the Cas9(D10A) nickase, *R*^2^ = 0.51, *p* = 0.045 for the H840A nickase) between indel formation and HDR frequencies with nickases, but no significant correlation with Cas9 nuclease (*R*^2^ = 0.08 *p* = 0.475) (Supplementary Fig. 2a). Although the absolute frequencies of HDR were 2.0-fold to 2.5-fold higher with Cas9 nuclease than with either Cas9 nickase (average across eight sites of 10% HDR product for Cas9, 5.0% for Cas9(H840A), and 4.0% for Cas9(D10A)), the HDR:indel ratio was 9.1-fold to 9.6-fold higher when using a nickase than Cas9 nuclease (the average HDR:indel ratio was 0.23 for Cas9, 2.1 for H840A, and 2.2 for Cas9(D10A)) (Fig. 1f). Importantly, we did not detect HDR above a frequency of 0.2% when dCas9 was paired with the same sgRNAs and donor templates (Fig. 1e), indicating that observed HDR frequencies are strongly dependent on Cas9 nicking, and are not artifacts of the donor template acting as a primer during the PCR reaction prior to HTS, a source of artificially high apparent HDR frequencies (Supplementary Fig. 7). To ensure that the donor templates did not participate in the PCR reactions used a size-selective DNA purification step (see Methods section and Supplementary Fig. 7). These experiments establish that nick-induced HDR results in improved HDR:indel ratios compared to DSB-mediated HDR. However, the unpredictable nature of whether a nickase will be able to mediate HDR at a particular locus, as well as generally low efficiency, limits the utility of simple nickase-mediated HDR.

### Modulating HDR by manipulating cellular repair proteins

To address these limitations, we sought to better understand the cellular proteins involved in catalyzing nick-induced HDR. To date, several studies have manipulated cellular DNA repair processes to favor HDR over NHEJ^17,22,24,36–38^. Previous efforts have identified key cellular DNA repair modulators that can be inhibited (such as p53 binding protein 1 (53BP1))^36,37^ or overexpressed (such as Rad52^36^) to improve HDR:indel ratios in response to a targeted DSB. Knockdown of cellular hRad51, or inhibition of hRad51 by overexpression of the dominant negative mutant hRad51(K133R), increases both indel and HDR frequencies at targeted nicks^17,22,24^. Guided by these observations, we chose to manipulate DNA repair modulators and study the resulting effects on DSB and nick-induced HDR.

**Fig. 2:**
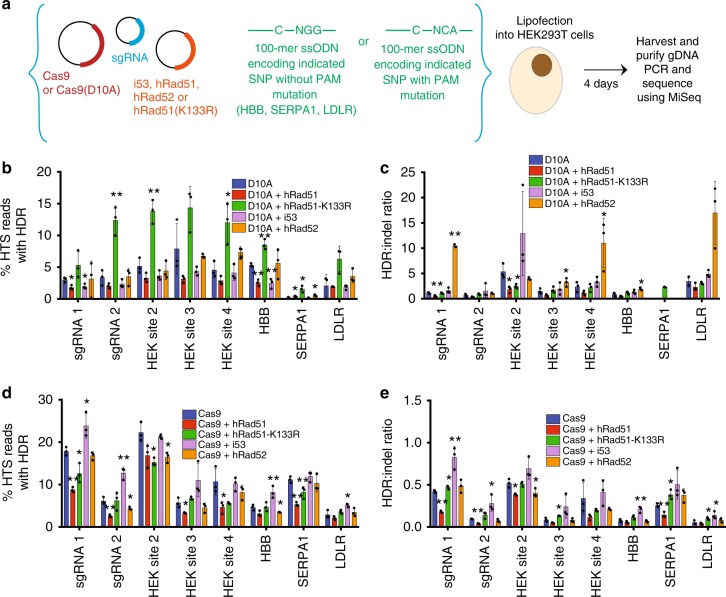
Manipulation of HDR frequency by global manipulation of cellular repair proteins. **a** Overview of experimental procedure. **b**, **d** HDR frequencies, measured by high-throughput DNA sequencing of unsorted HEK293T cells at eight endogenous genomic loci. **c**, **e** HDR:indel ratio at eight loci. **b**, **c** Data associated with treatment of Cas9(D10A) nickase and **d**, **e** with Cas9 nuclease. All data are shown as individual data points and mean ± s.d. for *n* = 3 independent biological replicates, performed on different days. Students two-tailed *t*-test was used to determine statistical significance between the indicated sample and in **b** Cas9(D10A) alone or in **d** Cas9 alone. (*): 0.01 < *p* < 0.05; (**): 0.001 < *p* < 0.01. Source data are provided in the Source Data file

We overexpressed either human hRad51 or hRad51(K133R) in conjunction with Cas9 or the Cas9(D10A) nickase (Fig. 2a). Overexpression of hRad51 led to a significant (*p* < 0.05; Student’s two-tailed *t*-test, Supplementary Table 4) decrease in HDR frequency at two of eight tested loci for Cas9(D10A) nick-mediated HDR (Fig. 2b) and at five of eight loci for DSB-mediated HDR (Fig. 2d). Conversely, overexpression of hRad51(K133R), which inhibits cellular hRad51 activity, led to an increase in the efficiency of nick-induced HDR, but not DSB-induced HDR (Fig. 2b, d). Finally, HDR:indel ratios remained largely unchanged by overexpression of hRad51 or hRad51(K133R). Together, these data demonstrate that hRad51 inhibition increased both HDR and indel frequencies at nick sites, but not at Cas9-induced DSBs (Fig. 2c, e). Intriguingly, overexpression of hRad51(K133R) led to low but detectable levels of HDR at the previously refractory *SERPA1* site (Fig. 2b).

To test the potential effect of p53 binding protein 1 (53BP1) on nick-induced HDR, we overexpressed i53, a protein inhibitor of 53BP1^37^. 53BP1 directs DSBs towards NHEJ-mediated repair by preventing end resection, a key event on the HDR pathway^39^. Overexpression of i53 with Cas9 led to a significant (defined as *p* < 0.05, Student’s two-tailed *t*-test) increase in the absolute frequency of HDR at four of eight tested loci and an improvement in the HDR:indel ratio at six of eight loci compared to Cas9 alone (Fig. 2d, e, Supplementary Tables 4 and 5). No such HDR improvements from i53 overexpression were observed, however, when Cas9(D10A) nickase was used instead of Cas9 (Fig. 2b, c), indicating that 53BP1 is unlikely to be a key modulator of nick-mediated HDR. Overexpression of Rad52, an interaction partner of hRad51, did not increase the efficiency of HDR arising from nicks or DSBs, but significantly improved the HDR:indel ratio at four of eight loci when HDR was stimulated by a nick (Fig. 2b–e). Together, these findings suggest that global inhibition of cellular hRad51, but not inhibition of 53BP1 or elevating Rad52 levels, can increase the frequency of HDR in response to a DNA nick.

### Development of Cas9(D10A)nickase fusions that promote HDR

Based on the above findings, we generated fusion constructs between the Cas9(D10A) nickase or the Cas9(H840A) nickase and hRad51(K133R) to mediate local inhibition of hRad51 at the target site. We anticipated that such fusion constructs might be more effective and less perturbative than global inhibition of hRad51, which causes chromosomal instability^40^, and that the hRad51 fusion partner would serve to modulate repair of the DNA nick. Under normal circumstances, cellular hRad51 binds to exposed genomic ssDNA after end-resection at the nick, leading to perfect, non-mutagenic repair of the nick^13,22^. This non-mutagenic repair process is inhibited by the dominant negative hRad51(K133R) mutant, which forms mixed filaments with wild-type hRad51 that can perform a DNA homology search, but cannot hydrolyze ATP to initiate DNA strand invasion^41^, even when low levels of the mutant protein are present^42^.

**Fig. 3:**
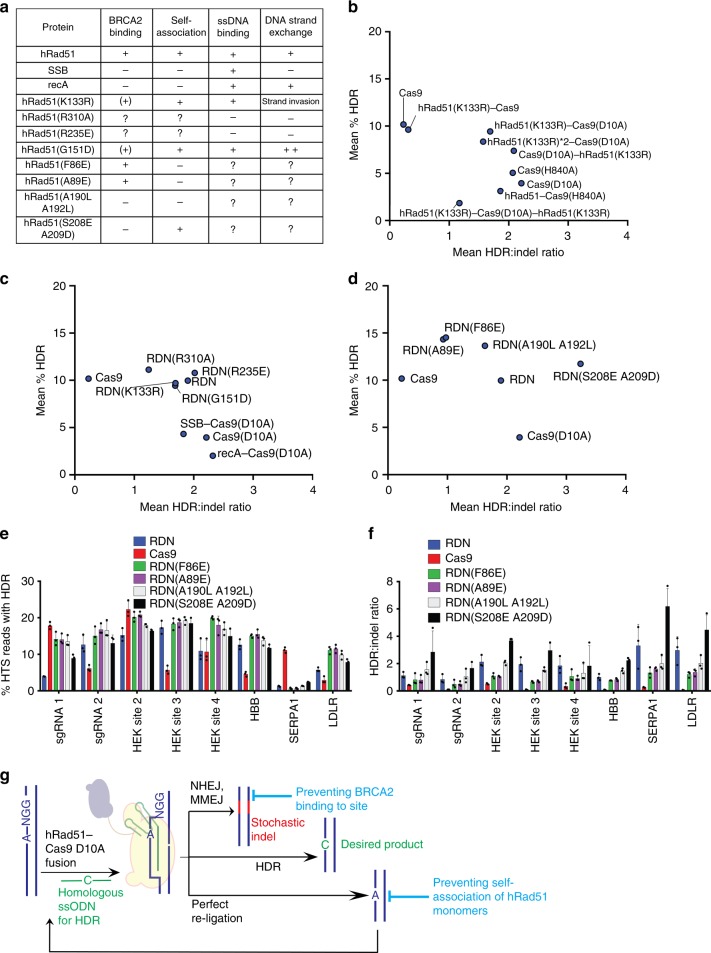
HDR frequencies associated with fusion constructs between hRad51 and its mutants and Cas9 or Cas9 nickases. **a** Catalytic activity and protein-protein binding interactions associated with hRad51, mutants of hRad51 and the homologous protein recA. + indicates activity has been validated; – indicates the absence of activity has been validated; ? indicates activity is unknown; (+) indicates activity has not been explicitly validated but is expected from structural data; and ++ indicates improved activity relative to wild type. **b**–**d** Dot plots depicting the average HDR frequencies and the average HDR:indel ratio associated with the indicated construct measured by high-throughput sequencing in unsorted HEK293T cells at eight loci. **b** Comparison between fusion constructs between Cas9(D10A) and hRad51(K133R) with different fusion architectures. **c** Comparison between catalytic mutants of hRad51 bound to the N-terminus of Cas9(D10A). **d** Comparison between binding mutants of hRad51 bound to the N-terminus of Cas9(D10A). **e** HDR frequencies associated with hRad51 and the mutants depicted in **d**, plotted by genomic locus. **f** HDR:indel ratio associated with editing at eight loci. For **e**, **f**, data are shown as individual data points and mean ± s.d. for *n* = 3 independent biological replicates, performed on different days. **g** Model of possible editing outcomes from hRad51–Cas9(D10A) nickase fusions. Source data are provided in the Source Data file

We began by optimizing the parameters for transfection by performing a titration of plasmid and donor template quantities and by measuring HDR and indel efficiencies at two loci with both the Cas9(D10A) nickase and the hRad51(K133R)–Cas9(D10A) fusion (Supplementary Figs. 3a–d). To our surprise, a small quantity of ssODN (50 ng) was sufficient for efficient HDR, and increasing the ssODN amount to 400 ng reduced HDR efficiency. Fusion of hRad51(K133R) to the N-terminus of the Cas9(D10A) nickase increased HDR efficiency in HEK293T cells by an average of 2.4-fold without altering the favorable HDR:indel ratio observed with the Cas9(D10A) nickase alone (Fig. 3b). We refer to this fusion construct, hRad51(K133R)–Cas9(D10A) nickase, as RDN(K133R). Compared to RDN(K133R), moving the position of hRad51(K133R) to the C terminus of the Cas9(D10A) nickase did not significantly alter HDR frequencies (Fig. 3b), nor did fusing an additional monomer of hRad51(K133R) to the N-terminus of Cas9(D10A) (Fig. 3b). Fusion of one hRad51(K133R) monomer to the N-terminus and one to the C-terminus, however, reduced both HDR and indel formation, possibly due to the association of multiple fusion proteins into an extended multimer (Fig. 3b). Consistent with the data showing that inhibition or overexpression of hRad51 does not have a substantial effect on DSB-mediated HDR, fusion between Cas9 and hRad51(K133R) led to a slight reduction to average HDR frequency at the loci tested (Fig. 3b; Supplementary Figs. 4a, b). Fusion between hRad51(K133R) and the Cas9(H840A) nickase also did not improve HDR frequency or HDR:indel ratios (Supplementary Figs 5e and 5f). The nickase strand preference of HDR enhancement upon hRad51(K133R) fusion may arise from the position of the nick introduced by Cas9(H840A) in the R-loop of displaced genomic DNA, compared with the position of the nick from Cas9(D10A) in the DNA:RNA duplex (Fig. 1b).

Surprisingly, fusion of wild-type hRad51 to Cas9(D10A), hereafter referred to as RDN, also resulted in increased HDR efficiency (Fig. 3c), even though overexpression of hRad51 *in trans* with the Cas9(D10A) nickase lead to slightly decreased HDR efficiency (Fig. 2b). These results suggest that increased HDR frequency mediated by RDN results from a mechanism distinct from global inhibition of hRad51. Together, these data demonstrate that localizing hRad51 to a targeted DNA nick through the RDN fusion increases nick-mediated HDR efficiency without inhibition of strand invasion mediated by cellular hRad51.

Next, we sought to understand if the HDR frequency enhancement associated with RDN and RDN(K133R) arises from simple steric occlusion of DNA repair proteins from accessing the nick, or whether the affinity of hRad51 for single-stranded DNA leads to localization of the single-stranded DNA donor to the nick. To illuminate these possibilities, we created fusions between the Cas9(D10A) nickase and RecA or bacteriophage T4-derived single-stranded binding protein (SSB). RecA is a bacterial homolog of hRad51 that catalyzes strand invasion between homologous strands of DNA. Neither RecA–Cas9(D10A) nor SSB–Cas9(D10A) resulted in HDR enhancement (Fig. 3c). Furthermore, incorporation of three additional hRad51 mutants (R310A, R235E and G151D) into RDN to generate RDN(R310A), RDN(R235E) and RDN(G151D) all displayed HDR enhancement frequencies indistinguishable from that of RDN and RDN(K133R) (Fig. 3c, and Supplementary Figs. 5i, j), in spite of their differing catalytic and DNA-binding characteristics (Fig. 3a)^43–45^. Taken together, these observations reveal that neither the fusion orientation of hRad51 relative to Cas9(D10A) nor the strand invasion and strand exchange activities of hRad51 are critical for the ability of RDN to mediate HDR.

### Donor template optimization

When possible, including a PAM-altering mutation together with the target mutation in a donor template is an effective approach to improve HDR efficiency^7,46^ by preventing re-cutting and subsequent modification of the desired HDR product. HDR efficiencies are highly dependent on the distance between the DNA cleavage site and the mutation that is being incorporated^7,46^. The above experiments used donor templates that contain PAM-blocking mutations at five of the eight loci tested (sgRNA 1, sgRNA 2, HEK site 2, HEK site 3, and HEK site 4), and donor templates that lacked PAM-blocking mutations due to unavailability of a silent PAM-blocking mutation in addition to the target point mutation at the remaining three sites (LDLR, HBB, and SERPA1). Since indels are generated much less efficiently with nick-induced HDR compared to DSB-induced HDR (Fig. 1e), we sought to test whether PAM-blocking mutations are necessary for nick-induced HDR and to define the region between the PAM and target mutation that can support efficient HDR.

**Fig. 4:**
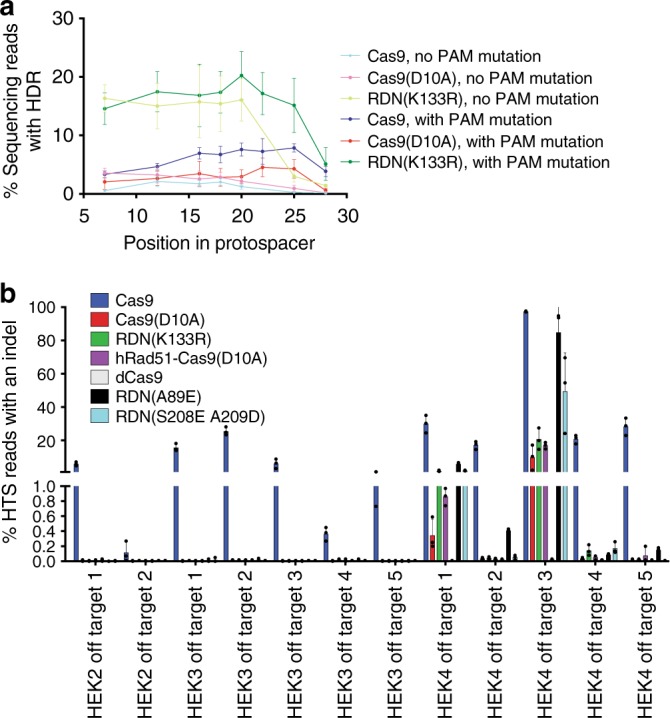
Characterization of positional dependence and off-target editing of nick-mediated HDR. **a** HDR frequencies measured by high-throughput sequencing in unsorted HEK293T cells using ssODNs with point mutations distributed along the sgRNA protospacer sequence of the HEK 3 sgRNA site. In previous figures, an oligonucleotide with a different PAM-blocking mutation at HEK Site 3 was used to measure an SNP incorporated at position 12 in the protospacer. **b** Indel frequencies at off-target genomic loci in cells treated with Cas9 nuclease, Cas9(D10A) nickase, or Cas9(D10A) fusions with hRad51 or the indicated mutants thereof. Dead Cas9 (dCas9) treated cells were included as a negative control. All data are shown as individual data points and mean ± s.d. for *n* = 3 independent biological replicates, performed on different days. Source data are provided in the Source Data file

We designed a series of eight ssODN templates targeting the HEK site 3 locus, each containing a SNP located in a different position within the protospacer from position 7 to 25, counting the PAM as positions 21–23. Two sets of donor templates were used. The first set of ssODNs incorporated a PAM mutation (replacing the TGG PAM with TTT) alongside the target mutation, while the second set only encoded each target mutation. As expected, we observed an increase in the frequency of Cas9-mediated HDR when the PAM-blocking template was used compared to the non-PAM-blocking template (Fig. 4a). By contrast, incorporating a PAM mutation into the donor ssODN did not lead to increased HDR frequency for nick-induced HDR, mediated either by Cas9(D10A) or RDN(K133R), as long as the target mutation is located within the sgRNA protospacer sequence (Fig. 4a).

We previously measured the frequency of HDR at HEK site 3 using a donor template with a PAM-blocking mutation (replacing the TGG PAM with TCC, Supplementary Tables 1 and 2) using Cas9 (Fig. 1e), Cas9(D10A) (Fig. 1e), or RDN(K133R) (Supplementary Fig. 5c). The HDR frequencies from Cas9 and RDN(K133R) were very similar when these different oligonucleotides were used. For example, Cas9 yielded 4.7 ± 0.5% HDR with a TTT-blocking mutation, and 5.7 ± 0.9% with a TCC-blocking mutation. However, the mean value for Cas9(D10A) increased from 2.6 ± 1.0% with the TCC PAM blocking mutation to 7.9 ± 3.2% with the TTT PAM blocking mutation, an unexpected result that suggests some ssODN dependence for Cas9(D10A)-mediated HDR.

Unlike DSB-induced HDR, in which HDR efficiency steeply declines as the distance between the DSB and the incorporated mutation increases^7,46^ (Fig. 4a), we observed comparable HDR efficiencies when RDN(K133R) was paired with different donor templates that introduced mutations from position 7 to 18 in the protospacer (Fig. 4a). This greater apparent independence of HDR efficiency from the location of the mutation to be installed relative to the protospacer suggests that RDN may offer more flexibility with regards to guide RNA choice than Cas9 nuclease-mediated HDR.

We also tested donor template oligonucleotides that were oriented in the same sense as the sgRNA (forward template, which was used for all other experiments in this study) and in the opposite sense (reverse template). We did not observe any significant differences (Student’s two-tailed *t*-test) in the resulting HDR efficiencies mediated by Cas9(D10A), Cas9, Cas9(H840A), or RDN(K133R) (Supplementary Fig. 4c), indicating that ssODN orientation is not a substantial determinant of HDR efficiencies under the conditions tested.

### RDN with additional hRad51 mutants

Although the development of RDN as a tool to mediate HDR led to consistently improved HDR:indel ratios, the overall frequency of RDN-mediated HDR is similar to that of Cas9-mediated HDR (Fig. 3c). In an attempt to improve overall HDR efficiency further while maintaining favorable HDR:indel ratios, we assessed four additional mutants of hRad51 in RDN constructs.

In addition to their role in catalyzing DNA strand invasion, hRad51 monomers directly bind to BRCA2^47–49^, or to other hRad51 monomers^25,50^. Mutants of hRad51 that have lost either or both of these capabilities have been engineered^25,26^ (Fig. 3a). We installed these mutations into the RDN context and assayed HDR and indel outcomes of the resulting constructs to assess whether these binding interactions influence editing outcomes (Fig. 3d–f). The results revealed that using hRad51 mutants incapable of self-association, but which maintain BRCA2 binding, increased HDR efficiency in HEK293T cells at the eight tested sites to an average of 14% (F86E mutant, RDN(F86E)) or 15% (A89E mutant, RDN(A89E)), compared to 10% for RDN. Both of these mutants were associated with a modest reduction in HDR:indel ratio, from an average of 1.9 for RDN to 0.93 for RDN(F86E) or 0.98 for RDN(A89E).

In contrast, removing the BRCA2-binding ability of hRad51 using the double mutant (RDN(S208E A209D)) only slightly improved HDR efficiency relative to RDN (to an average of 12%), but substantially improved the HDR:indel ratio (to 3.3), suggesting that abolishing recruitment of BRCA2 to the nick promotes more favorable HDR:indel partitioning. We should note that even with these improvements, the efficiency of nick-induced HDR remains more sgRNA-dependent than the efficiency of DSB-induced HDR. For example, pairing original or mutant RDN constructs with sgRNA SERPA1 leads to modest (<3%) HDR frequencies compared with Cas9 (11.1 ± 0.6%).

We tested a final hRad51 A190L A192L double mutant that lacks both BRCA2-binding and hRad51 self-association ability. RDN(A190L A192L) mediated HDR with an average efficiency of 14% and an HDR:indel ratio of 1.6, offering intermediate levels of HDR efficiency and HDR:indel ratio compared to the above RDN variants.

These analyses inform potential mechanisms by which RDN can mediate efficient HDR with favorable HDR:indel ratios. The data are consistent with a model in which self-association of hRad51 is important to maintain a high HDR:indel ratio but also limits HDR efficiency by promoting perfect repair of the DNA nick. In contrast, recruitment of BRCA2 to the nick site reduces the rate of perfect repair of the nick (Fig. 3g). For applications that benefit most from maintaining the highest possible HDR efficiency, RDN(A89E) is the most useful, whereas applications that require maximizing the HDR:indel ratio will benefit from use of the RDN (S208E A209D) variant.

### Off-target modification by RDN variants

Cas9 nuclease^51^ and Cas9-derived proteins such as base editors^3,5,52^ can induce off-target editing in an sgRNA-dependent fashion. We characterized off-target editing at known off-target sites associated with three well-studied sgRNAs^51^: HEK site 2, HEK site 3, and HEK site 4, which is a notoriously promiscuous sgRNA^53,54^. Although the homology required between the target genomic locus and the ssODN prevents significant off-target HDR products from being generated by Cas9 combined with a ssODN, indel formation from Cas9 nuclease activity at off-target sites under these conditions is common. Off-target indel formation was measurable (>0.1%) with Cas9 treatment at all tested Cas9 off-target sites, and off-target indel formation ranged in efficiency from 0.12 to 98% (Fig. 4b). In contrast, Cas9(D10A) nickase and RDN edited only two of the 12 off-target loci (>0.1% indel formation) (Fig. 4b). The more efficient RDN(A89E) edited four of 12 off-target sites at a frequency >0.1%, all of which are associated with the promiscuous HEK site 4 guide RNA. These results indicate that RDN-mediated HDR offers substantially lower off-target DNA modification than nuclease-based HDR, and that this trend even applies to RDN(A89E), which typically results in higher on-target HDR frequencies than Cas9.

### HDR in other human cell types

HEK293 and HEK293T cells are known to be particularly amenable to ssODN-mediated HDR^55^. Indeed, some other commonly used immortalized cell lines including HeLa and U2OS are thought to be completely refractory to ssODN-mediated HDR^55^. We compared RDN- and Cas9-mediated HDR outcomes in other immortalized cell lines and in primary human cells, including HeLa cells, U2OS cells, human induced pluoripotent stem (hiPS) cells and K562 cells.

In HEK293T cells, RDN(A89E) offers the highest HDR frequency (Fig. 3e) and RDN(S208E A209D) offered the highest HDR:indel ratio (Fig. 3f) of all the constructs tested, so we tested these two constructs in the wider range of cell types. For this comparison, we used oligonucleotides designed without PAM mutations to maximize the generality of the results and due to our conclusions that nick-mediated HDR does not benefit from PAM blocking mutations (Fig. 4a). Unless otherwise specified, we report results from unsorted cells as percentages of the entire cell population, not as percentages of edited or modified cells, which would greatly increase apparent editing efficiencies.

**Fig. 5:**
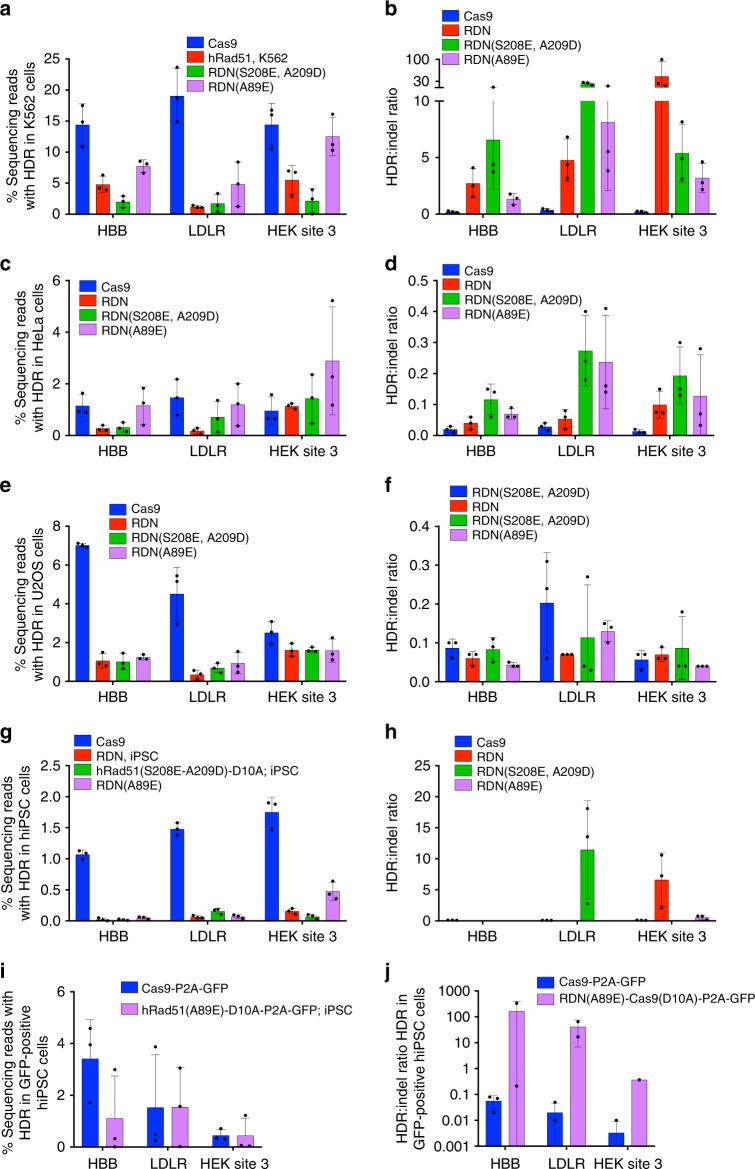
hRad51–Cas9(D10A) nickase activity in K562, U2OS, HeLa and hiPS cells. **a**, **c**, **e**, **g** HDR frequencies, measured by high-throughput sequencing in unsorted, nucleofected cells at three loci; **b**, **d**, **f**, **h** HDR:indel ratio associated with editing at the same three loci. **i**, **j** HDR frequency and HDR:indel ratios in iPS cells nucleofected with P2A-GFP tagged constructs and sorted for GFP-positive cells. All data are shown as individual data points and mean ± s.d. for *n* = 3 biological replicates, performed independently. An HDR:indel ratio was not reported if the HDR frequency was <0.1% (see Methods section). Source data are provided in the Source Data file

RDN (containing wild-type hRad51) led to substantially reduced HDR frequencies when compared to Cas9 in all non-HEK293T cell types tested. For example, in K562 cells the average reduction in efficiency was from a mean of 16% with Cas9 to 3.8% with RDN (Fig. 5a). The mean HDR:indel ratio, however, was improved 87-fold in K562 cells and 3-fold in HeLa cells (Fig. 5b, d). RDN(S208E A209D) demonstrated slightly improved HDR:indel ratios when compared to RDN, but the overall efficiency of HDR remained low compared to that achieved by Cas9 (Fig. 5).

When RDN(A89E) was used, however, the average HDR efficiency was substantially improved, with mean HDR frequencies of 8.3% in K562, 1.3% in U2OS, and 1.8% in HeLa cells (Fig. 5a, c, e). HDR efficiencies in these three non-HEK293T cell types were on average 2.1-fold lower than those following Cas9 treatment. RDN(A89E) was associated with a 15-fold improvement in HDR:indel ratio in K562 cells and 7-fold in HeLa cells compared to Cas9 treatment. This improvement was not observed in U2OS cells, which exhibited a slight reduction in HDR:indel ratio when RDN(A89E) was used (Fig. 5f). In hiPS cells, only one of the three tested loci was amenable to RDN(A89E)-mediated HDR, demonstrating that this approach may be more site-dependent in hiPS cells than in immortalized cell lines. To test if this limitation was due to poor expression of RDN(A89E) in hiPS cells, we generated Cas9 and RDN(A89E) constructs tagged with P2A GFP to enable isolation of Cas9-expressing or RDN(A89E)-expressing cells. With Cas9–P2A–GFP, isolating GFP-positive cells resulted in 1.8% average HDR efficiencies in hiPS cells with an average HDR:indel ratio of 0.03 (Fig. 5i, j). Among GFP-positive cells expressing RDN(A89E)–P2A–GFP, average HDR efficiencies were 1.0%, with an average HDR:indel ratio of 46 (Fig. 5i, j), reflecting a modest decrease in HDR efficiency but a >1000-fold improvement in HDR:indel ratio. (Fig. 5i, j, Supplementary Fig. 8). Among GFP-positive cells isolated with the RDN(A89E)-P2A-GFP construct, average indel frequency was 1.6% and the vast majority showed no target site modification. This observation suggests that the majority of nicks induced by RDN(A89E) construct are perfectly repaired in hiPS cells; in contrast, GFP-positive cells containing Cas9–P2A–GFP contained an average of 77% indels.

These data together reveal that RDN(A89E) mediates more efficient HDR than Cas9 nuclease in HeLa and HEK293T cells, maintains similar levels of HDR efficiency in K562 cells, and offers improved HDR:indel ratios in HeLa, HEK293T, K562, and hiPS cells. Neither an efficiency nor a product purity advantage from any tested RDN variant was observed in U2OS cells, possibly as a result of unusual regulation of DNA repair in U2OS cells^55,56^. This variability is likely due to the reliance of RDN on cellular repair processes that are highly cell type-dependent.

## Discussion

The method developed in this study enables precise and specific changes to be made to genomic DNA through homology-directed repair, without generating a double stranded DNA break. Use of the fusion construct hRad51–Cas9(D10A) (RDN) or variants of this construct in which hRad51 has been replaced by hRad51 mutants, can address some of the challenges associated with using HDR to make precise changes to genomic DNA in certain human cell types.

The HDR:indel ratio generated by RDN is generally improved compared to that which can be achieved using a DSB. This improvement in the purity of editing outcomes is particularly important for genome editing applications in which gene knockout resulting from indel formation opposes desired biological outcomes, or in which mixtures of many different edited genotypes—the typical cellular response to DSBs—is undesired. The RDN(S208E A209D) construct is particularly useful under such circumstances since it offers ~3.2-fold more HDR product than indels (Fig. 3d). In addition, the efficiency of HDR mediated by RDN and RDN(A89E) is higher than that of Cas9 in some (but not all) cell types (Figs. 3e, 5a), although HDR efficiency remains modest, likely limited by dependence on cellular DNA repair processes. RDN and its variants also offer substantially higher DNA specificity (lower off-target indel formation) compared to Cas9 nucleases combined with the same sgRNAs, even when applied to a notoriously promiscuous guide RNA with many known off-target loci (Fig. 4b). RDN with wild-type hRad51 offers the greatest degree of DNA specificity among the mutants tested, but this difference was only notable at the promiscuous HEK Site 4, as were not able to detect off-target editing at frequencies above 0.2% at any other tested loci following use of RDN, RDN(A89E) or RDN(S208E A209D) (Fig. 4b). Finally, since RDN variants cannot directly generate DSBs, we anticipate that the likelihood of inducing translocations, large deletions, or p53 activation will be greatly reduced compared to nuclease-based genome editing methods. Additional studies using are needed to fully characterize the scope of cellular responses to targeted nicks compared to targeted DSBs.

We anticipate that RDN(A89E) or RDN(S208E A209D) will be useful for applications in which efficiency or cleanliness of genome editing are critical. Recent work whereby saturation genome editing was performed to investigate variants of unknown significance in *BRCA1*^57^ highlight the utility of a tool with the ability to generate mutations with single nucleotide resolution. Nuclease-mediated approaches to saturation editing can only be performed on essential genes because of the requirement that cells in which indels are induced must be excluded from the analysis. The favorable HDR:indel ratio and HDR efficiency offered by RDN may permit mutagenesis with nucleotide-level resolution on non-essential genes. Finally, we hope that the principles illuminated in this work will be enabling for researchers seeking to develop new methods for studying and manipulating cellular DNA damage and repair.

## Methods

### Plasmid cloning

All mammalian cell expression plasmids were constructed by USER cloning from gBlock gene fragments (Integrated DNA Technologies) with USER junctions sized between 14 and 20 nucleotides^58^. Phusion U Green Multiplex PCR Master Mix (ThermoFisher) was used for amplification of DNA. sgRNA plasmids were constructed by blunt end ligation of a linear PCR product generated by encoding the 20-nt variable protospacer sequence onto the 5′ end of an amplification primer and treating the resulting piece to KLD Enzyme Mix (New England Biolabs) according to the manufacturers’ instruction. Mach1 chemically competent *E. coli* (ThermoFisher) cells were used.

### Preparation of plasmids for mammalian cell transfection

To obtain endotoxin-free plasmids for transfection, 45 mL of Mach1 cells expressing freshly-transformed plasmid were pelleted by centrifugation (6000×*g*, 10 min, 4 °C) and purified using ZymoPURE II Plasmid Midi Prep Kits (Zymo Research), according to the manufacturer’s instructions with the inclusion of the optional step of passing the plasmid across the EndoZero Spin column (Zymo Research). Plasmid yield was quantified using a Nanodrop and by electrophoresis on a 1% agarose Tris/Borate/EDTA gel supplemented with ethidium bromide.

### Mammalian cell culture

All cells were cultured and maintained at 37 °C with 5% CO_2_. Antibiotics were not used for cell culture. HEK293T cells (ATCC CRL-3216) and HeLa cells (ATCC CCL-2) were cultured in Dulbecco’s modified Eagle’s medium (DMEM) plus GlutaMax (ThermoFisher) supplemented with 10% (v/v) fetal bovine serum (FBS). K562 cells (ATCC CCL-243) were cultured in Roswell Park Memorial Institute (RPMI) 1640 Medium plus GlutaMax (ThermoFisher) supplemented with 10% (v/v) FBS. U2OS cells were cultured in MyCoy’s 5A Medium plus GlutaMax (ThermoFisher) supplemented with 10 % (v/v) FBS.

hiPS cells (human episomal iPS cell line; A18945; ThermoFisher) were cultured in Essential 8 Flex Medium (ThermoFisher) supplemented with RevitaCell after passaging (ThermoFisher) according to the manufacturer’s directions. Versene (Thermo Fisher) was used for cell passaging and dissociation. Prior to nucleofection, cells were harvested with Accutase (ThermoFisher).

For data shown in Fig. 5i, j, nuclease expression plasmids were constructed whereby the Cas-enzyme construct (Cas9 or RDN(A89E)) was proceeded by P2A-GFP to enable isolation of transfected cells. iPS cells were flow sorted at the MIT FACS core 3–5 days after nucleofection and genomic DNA was isolated directly after sorting.

### Mammalian cell lipofection and genomic DNA isolation

HEK293T cells were seeded on 48-well poly-d-lysine coated plates (Corning) 16–20 h before lipofection. Lipofection was performed at a cell density of 65%. Unless otherwise stated, cells were transfected with 231 ng of nuclease-editor or base-editor expression plasmid DNA, 69 ng of sgRNA expression plasmid DNA, 50 ng (1.51 pmol) 100-nt ssODN (PAGE-purified; Integrated DNA Technologies) and 1.4 µL Lipofectamine 2000 (ThermoFisher) per well. For experiments where global inhibition or overexpression of a cellular HDR-component was performed 100 ng of the appropriate plasmid was included. Cells were harvested 4 days post-transfection and genomic DNA isolation and purification was performed with Agincort DNAdvance Kit (Beckman Coulter), according to the manufacturer’s protocol. Size-selective DNA purification was necessary to prevent contamination of gDNA with donor ssODN HDR templates. For analysis of indel formation in Supplementary Fig. 1, HeLa and U2OS cells were transfected according to the above protocol except they were transfected at a density of 80% with 1.4 µL Lipofectamie 3000 and 1 µL of P3000 (ThermoFisher) per well.

### Nucleofection of mammalian cells

For data generated in Fig. 5, nucleofection of K562, HeLa and U2OS cells was performed. For these three cell types, 350 ng nuclease-expression plasmid, 150 ng sgRNA-expression plasmid and 200 pmol (6.6 μg) 100-nt ssODN (PAGE-purified; Integrated DNA Technologies) was nucleofected in a final volume of 20 µL per sample in a 16-well Nucleocuvette strip (Lonza). K562 cells were nucleofected using the SF Cell Line 4D-Nucleofector X Kit (Lonza) with 5 × 10^5^ cells per sample (program FF-120), according to the manufacturer’s protocol. U2OS cells were nucleofected using the SE Cell Line 4D-Nucleofector X Kit (Lonza) with 3–4 × 10^5^ cells per sample (program DN-100), according to the manufacturer’s protocol. HeLa cells were nucleofected using the SE Cell Line 4D-Nucleofector X Kit (Lonza) with 2 × 10^5^ cells per sample (program CN-114), according to the manufacturer’s protocol. Cells were harvested 48 hours after nucleofection; genomic DNA was purified using the Agincort DNAdvance Kit (Beckman Coulter), according to the manufacturer’s protocol.

hiPS cells were nucleofected with 400 ng nuclease-expression plasmid, 400 ng sgRNA-expression plasmid and 200 pmol (6.6 μg) 100-nt ssODN (PAGE-purified; Integrated DNA Technologies) in a final volume of 20 µL per sample in a 16-well Nucleocuvette strip (Lonza) using the CB-150 program in the P3 Primary Cell 4D-Nucleofector X Kit (Lonza) with 0.75–1.5 × 10^6^ cells per sample.

### Preparation of genomic DNA for high throughput sequencing

Sites of interest were amplified using the primers listed (Supplementary Table 3). Amplification primers for the first PCR reaction (PCR1) were designed with primer2 and had 5′. extensions to enable amplification with an Illumina barcoding primer in a second PCR reaction (PCR2). Phusion U Green Multiplex PCR Master Mix (ThermoFisher) was used for both PCR1 and PCR2. For PCR1, each reaction contained 0.5 μM of the appropriate forward and reverse primer (Supplementary Table 3) and 30–100 ng of genomic DNA was as a template. Cycling conditions were 98 °C for 1 min 30 s, then 30 cycles of (98 °C for 10 s, 61 °C for 15 s, and 72 °C for 15 s) followed by a final extension of 1 min at 72 °C per 30 µL reaction. PCR1 products were verified on a 2% agarose gel Tris/Borate/EDTA gel supplemented with ethidium bromide. For PCR2, 1 µL of unpurified PCR1 plus 0.5 μM of each of a unique forward and reverse barcoding primer pair were added to each sample for a final volume of 30 µL. Cycling conditions were 98 °C for 1 min 30 s, then 7 cycles of (98 °C for 10 s, 61 °C for 15 s, and 72 °C for 15 s) followed by a final extension of 1 min at 72 °C. PCR2 products were purified by gel electrophoresis on a 2% agarose gel using the QIAquick Gel Extraction Kit (Qiagen). Purified product was passed over a second Minelute column (Qiagen) for a further round of purification before quantification with QBit ssDNA HS Assay Kit (ThermoFisher) and sequenced using an Illumina MiSeq with 230–270-bp single end reads according to the manufacturer’s instructions.

### Analysis of HTS data

Demultiplexing of pooled sequencing reads was performed using the MiSeq Reporter software (Illumina). Crispresso-v2^35^ was used to perform alignments between sequenced amplicons and reference amplicons. Indels were quantified in a 10-bp window surrounding the expected cut site for each sgRNA. For quantification of HDR, we discarded reads that contained indels from the alignment to the reference sequence using “discard-indel-reads” filter. This approach ensured that we did not erroneously count reads that contained both an SNP incorporated through HDR and an indel as an HDR event, as has been previously described^7^. The resulting alignment contained only reads that do not contain an indel within the 10-bp window around the sgRNA cleavage site. Separately from the alignment matrix, the output of Crispresso-v2 reported the percentage of reads that had been excluded from the alignment because they contained an indel (%cells with indel). For each target point mutation that was incorporated via HDR, the alignment alone could be used to determine the % of non-indel containing cells (% indel-free cells with target mutation) that had successfully incorporated the target mutation. In order to assess the % of all cells that had the target mutation, the following correction was performed:

1 $${{\mathrm{\% }}\,{\mathrm{Cells}}\,{\mathrm{with}}\,{\mathrm{target}}\,{\mathrm{mutation}} = {\mathrm{\% }}\,{\mathrm{indelfree}}\,{\mathrm{cells}}\,{\mathrm{with}}\,{\mathrm{target}}\,{\mathrm{mutation}} \times \frac{{100{\mathrm{\% }} - {\mathrm{\%}}\,{\mathrm{cells}}\,{\mathrm{with}}\,{\mathrm{indel}}}}{{100{\mathrm{\% }}}}}$$

For calculation of HDR:indel ratio, the % cells with indel-free HDR at the indicated sequence was divided by the % cells with an indel in the 10-bp window surrounding the cleavage site. For experiments with HEK293T cells, where robust (>1%) HDR and indel percentages were detectable for many conditions, HDR:indel ratios were not calculated if HDR frequency was less than 1% for a particular sample, to avoid reporting artificially high HDR:indel ratios that could accompany very low frequency events. For the data shown in Fig. 5, HDR and indel frequencies were measured in cell types less able than HEK293T cells to support HDR. For these instances, an HDR:indel ratio was not reported if the HDR frequency was <0.1% for the same reason. For calculations in the text in which averages across sites were made, if an HDR:indel ratio was not calculated due to a low HDR rate, then the HDR:indel ratio was set to zero when calculating the mean to avoid artificially inflating HDR:indel ratios.

### Reporting summary

Further information on research design is available in the Nature Research Reporting Summary linked to this article.

## Supplementary information

Supplementary Information

Reporting Summary

Source Data

## Data Availability

Plasmids encoding the constructs used in this study are available on Addgene, accession numbers can be found in Supplementary Table 6. The accession numbers have been listed in Supplementary Table 6. The source data underlying Figs. 1c–f, 2b–e, 3b–f, 4a–b, and 5a-e and Supplementary Figs. 1a–b, 3a–d, 4, 5a–j, 6a–b, and 7a–b are provided as a Source Data file. High-throughput DNA sequencing data has been deposited in the NCBI Sequence Read Archive with BioProject accession number PRJNA515942 (SRP180368).
