# Supplementary material for: Development of hRad51–Cas9 nickase fusions that mediate HDR without double-stranded breaks
Source: Nat Commun. 2019 May 17;10:2212. doi: 10.1038/s41467-019-09983-4 (PMC6525190; doi:10.1038/s41467-019-09983-4)
Supplement: Supplementary file 1 — Supplementary Information [file 41467_2019_9983_MOESM1_ESM.pdf]

Development of hRad51–Cas9 nickase fusions that mediate HDR without double-stranded breaks

Holly A. Rees[1,2,3], Wei-Hsi Yeh[1,2,3,4], David R. Liu[1,2,3,*]

SUPPLEMENTARY INFORMATION

Supplementary Figure 1. Frequency of nick-induced indels in HeLa and U2OS cells. 2

Supplementary Figure 2. Correlation between HDR and indel frequencies and between indel frequencies and microhomology with Cas9 nuclease and Cas9 nickases. 3

Supplementary Figure 3. Titration of plasmid and ssODN quantities for lipofection-mediated transfection. 4

Supplementary Figure 4. Assessment of the effect of ssODN sense on HDR frequencies in HEK293T cells. 5

Supplementary Figure 6. Indel formation and base editing in HEK293T cells at the same genomic loci as shown in Figure 1. 7

Supplementary Figure 7. Comparison of apparent HDR frequencies with and without magnetic bead-based purification of genomic DNA. 8

Supplementary Figure 8. Gating examples for flow sorting human iPSC cells (hiPSC). 9

Supplementary Note 1. Indel formation and base editing arising from commonly used base editors at the genomic loci shown in Figure 1. 10

Supplementary Table 1. Single guide RNA (sgRNA) sequences and HDR products. 11

Supplementary Table 2. Donor template sequences used for HDR. 12

Supplementary Table 3. DNA primers used for amplification of genomic DNA prior to HTS. 13

Supplementary Table 4. P-values for comparisons between conditions for absolute HDR frequencies in HEK293T cells. 14

Supplementary Table 5. P-values for comparisons between conditions for HDR:indel ratios in HEK293T cells. 15

Supplementary Table 6. Accession numbers for plasmids deposited with Addgene. 16

Supplementary References 17

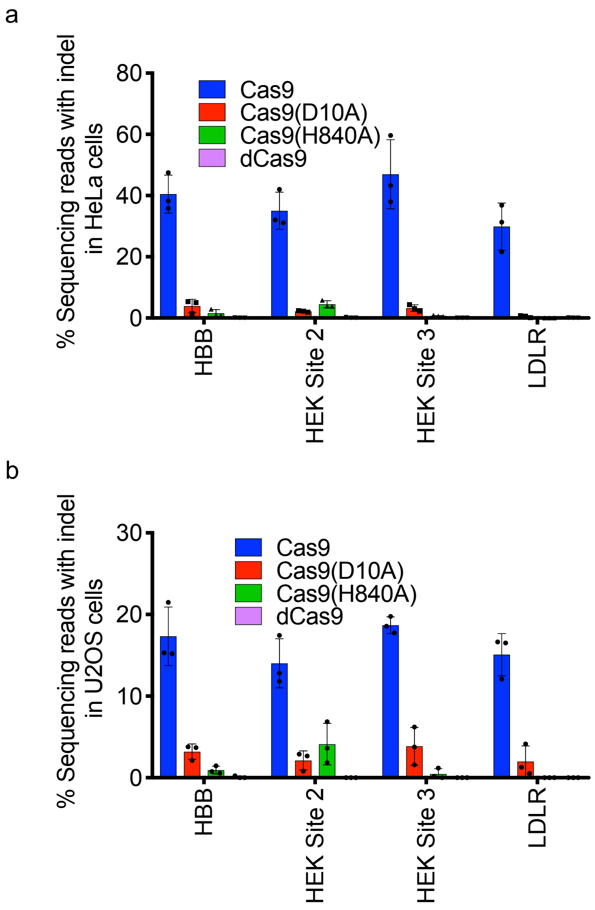

Supplementary Figure 1. Frequency of nick-induced indels in HeLa and U2OS cells. Cells were lipofected with Cas9, D10A, H840A nickase or dCas9 plasmid and a plasmid expressing the indicated sgRNA. DNA was harvested and sequenced from unsorted cells and subjected to HTS. **(a)** Indel frequencies in HeLa cells. **(b)** Indel frequencies in U2OS cells. All data are shown as individual data points and mean ± s.d. for n=3 independent biological replicates, performed on different days. Source data are provided in the Source Data file.

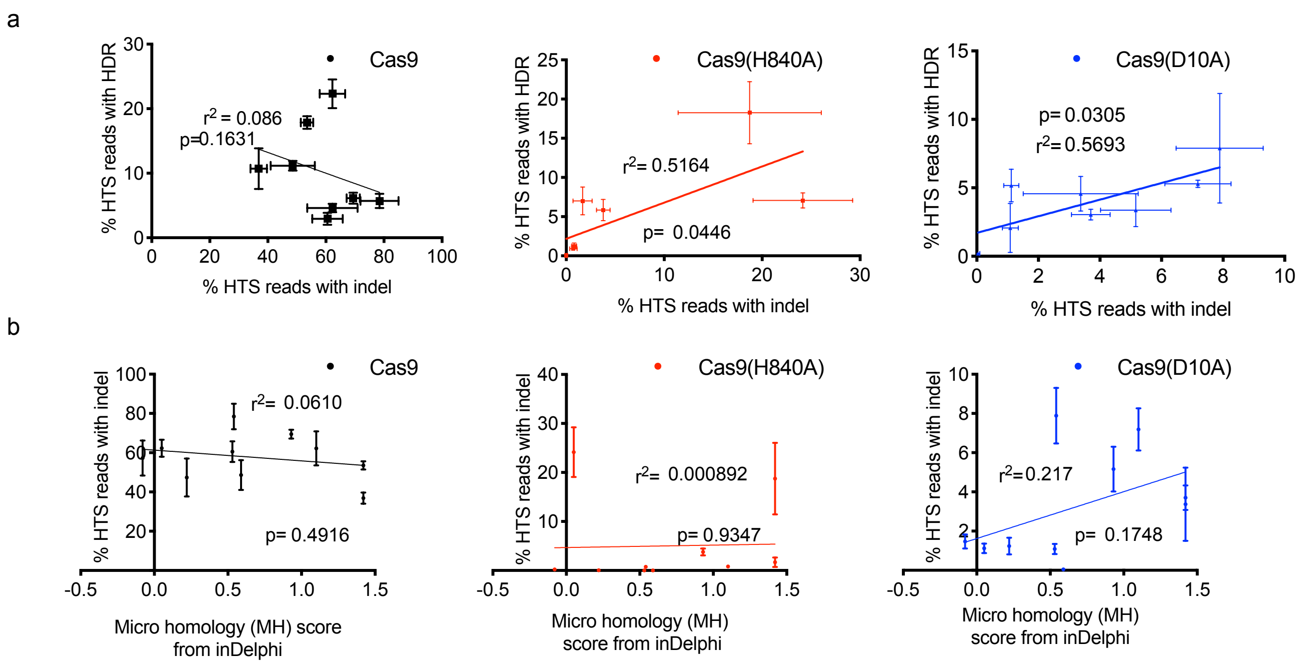

Supplementary Figure 2. Correlation between HDR and indel frequencies and between indel frequencies and microhomology with Cas9 nuclease and Cas9 nickases.
(a) Indel frequency in the absence of an ssODN plotted against HDR frequency. These data are also represented in Figure 1c and 1e. **(b)** Indel frequencies correlated to the micro homology score predicted by inDelphi[1] for each of the eight loci shown in Figure 1c and Figure 1d. For both **(a)** and **(b),** p-values were calculated in Prism by linear regression analysis. For **(a),** p-values represent a linear regression analysis to determine whether the slope is significantly non-zero. For **(b),** p-values represent a two-tailed test to determine whether the MH score is significantly correlated to the indel frequency. Data shown are the mean ± s.d. for n=3 independent biological replicates in HEK293T cells, performed on different days.

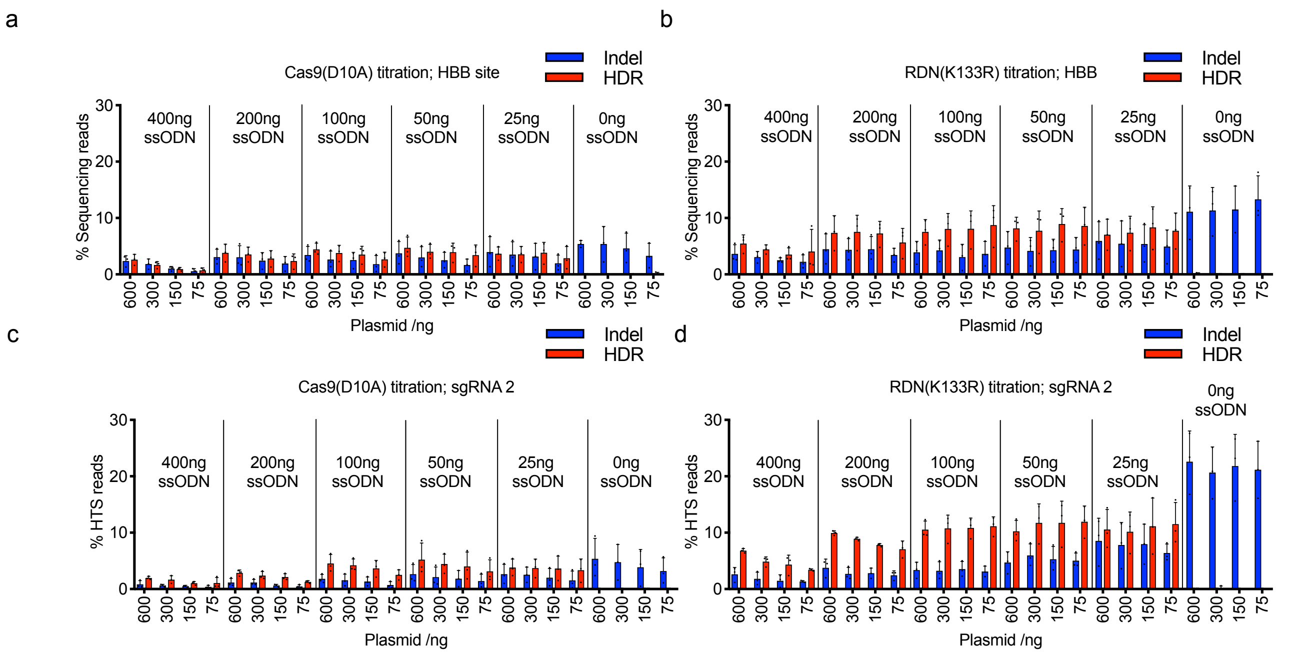

Supplementary Figure 3. Titration of plasmid and ssODN quantities for lipofection-mediated transfection.
HDR and indel frequencies associated with the indicated quantities of plasmid or ssODN, targeted to the indicated genomic locus. **(a)** and **(c)** show HDR and indel rates associated with D10A nickase; **(b)** and **(d)** show HDR and indel frequencies associated with the hRad51(K133R)-D10A fusion. 1.4 µL Lipofectamine 2000 was used for all conditions. All data are shown as individual data points and mean ± s.d. for n=3 independent biological replicates, performed on different days. Source data are provided in the Source Data file.

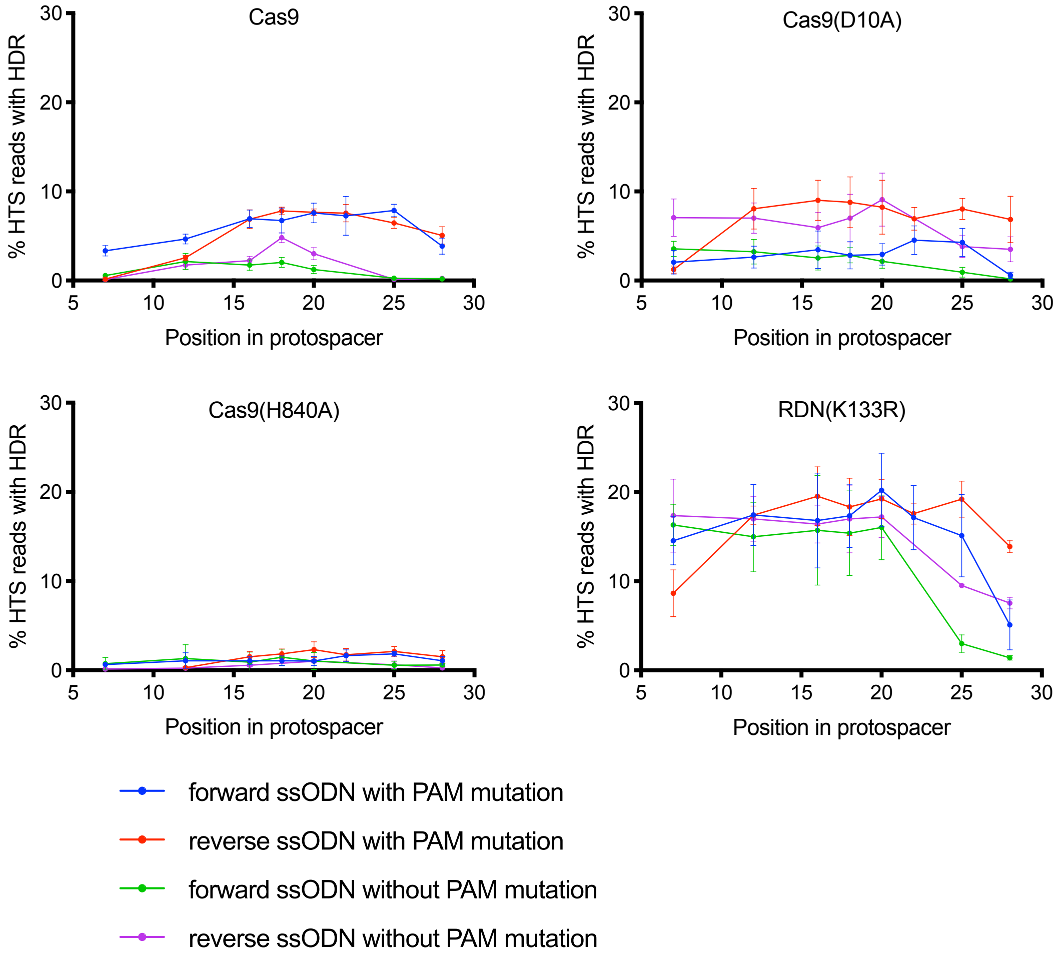

Supplementary Figure 4. Assessment of the effect of ssODN sense on HDR frequencies in HEK293T cells.
Related to Figure 4a. The single-stranded oligonucleotide donor (ssODN) sense (forward or reverse) was varied in the context of introducing single point mutations at different locations at the HEK 3 locus. Forward ssODN indicates that the ssODN donor is in the same sense as the sgRNA; Reverse ssODN indicates that the ssODN donor is in the reverse sense relative to the sgRNA (See Supplementary Table 2). Data are shown as mean ± s.d. for n=3 independent biological replicates, performed on different days. Source data are provided in the Source Data file.

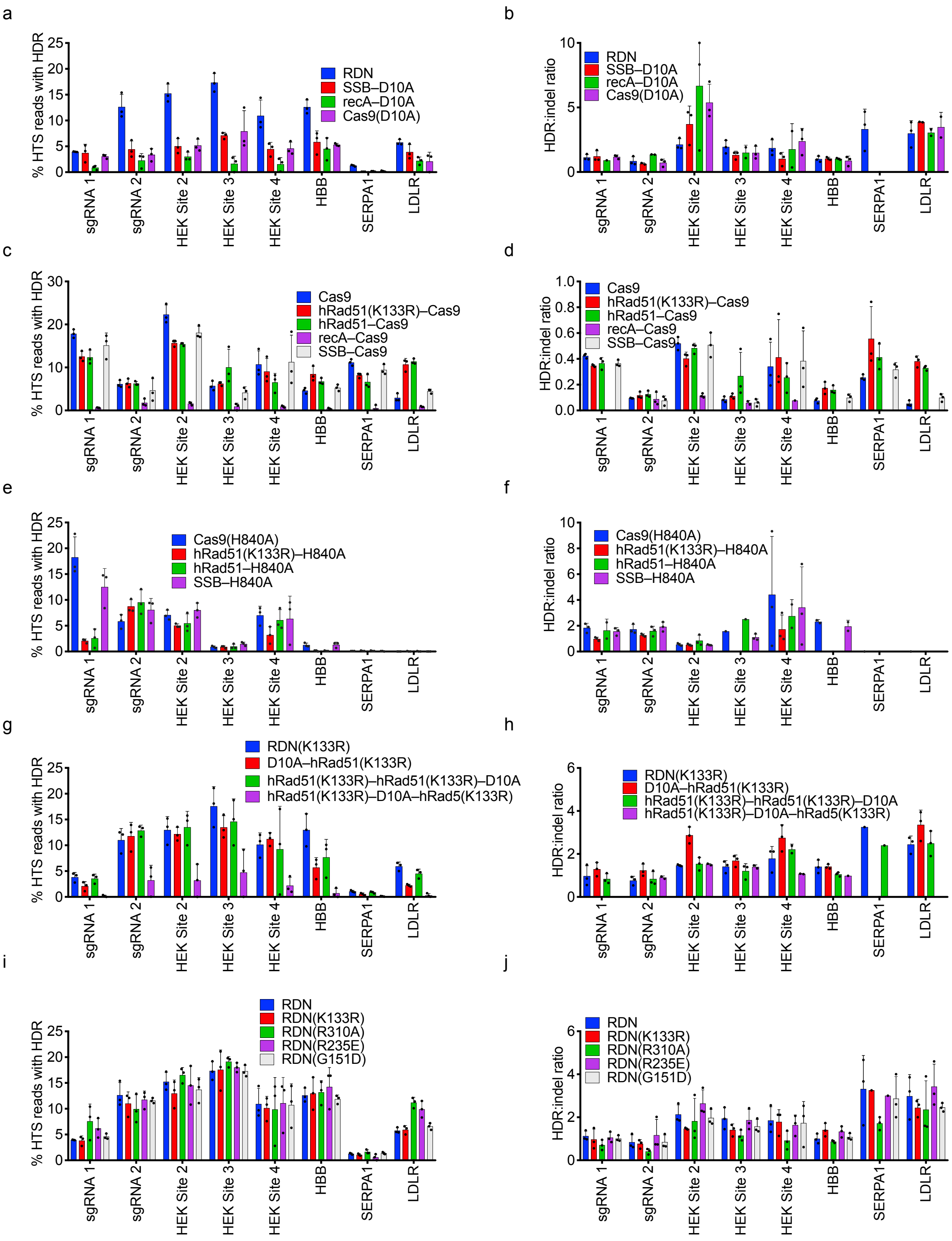

Supplementary Figure 5. Site-by-site plots of HDR frequency and HDR:indel ratios in HEK293T cells, as described in Figure 3.

(a), (c), (e), (g), (i) Site-by-site plots of HDR frequency. **(b), (d), (f), (h)** and **(j)** Site-by-site plots of HDR:indel ratio. These data are processed and plotted in Figure 3b and 3c. Data are shown as individual data points and mean ± s.d. for n=3 independent biological replicates, performed on different days. Note that RDN specifically refers to the construct containing an hRad51 monomer N-terminally fused to the Cas9(D10A) nickase (i.e. hRad51–Cas9(D10A)). Source data are provided in the Source Data file.

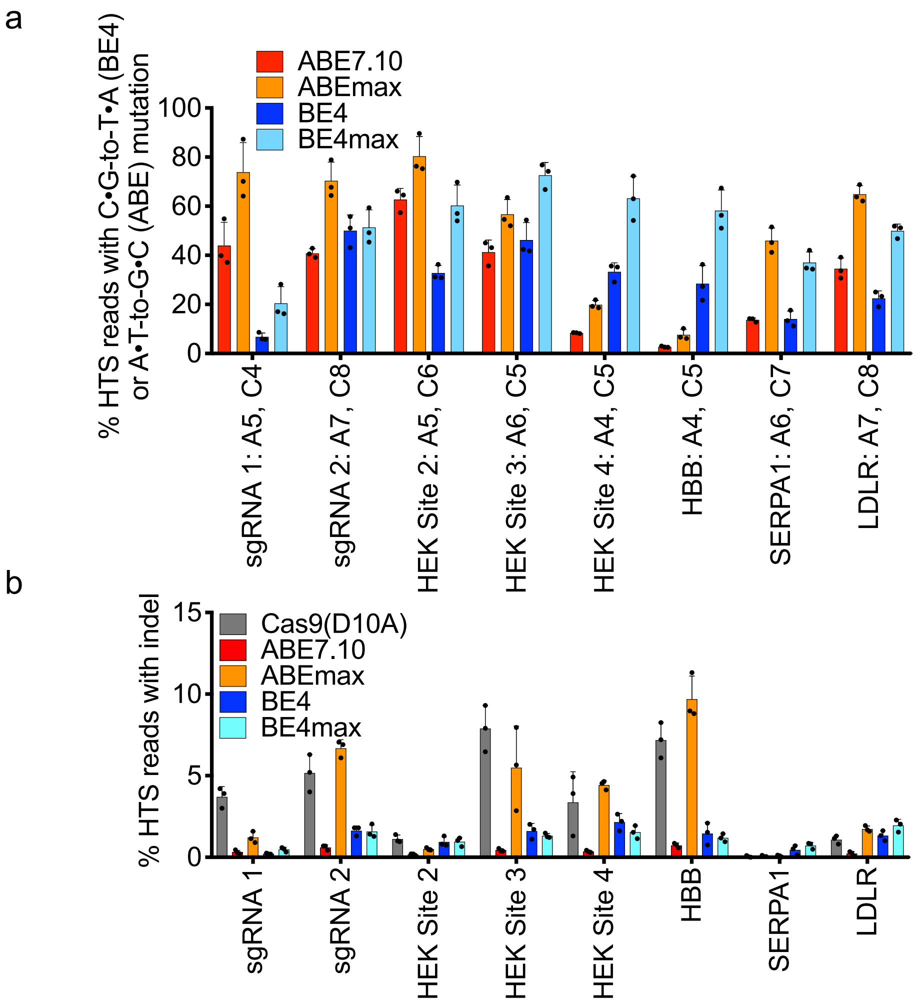

Supplementary Figure 6. Indel formation and base editing in HEK293T cells at the same genomic loci as shown in Figure 1.
(a) Indel frequencies associated with base editors and D10A nickase**. (b)** Base editing rates associated with base editors. All data are shown as individual data points and mean ± s.d. for n=3 independent biological replicates, performed on different days. Source data are provided in the Source Data file.

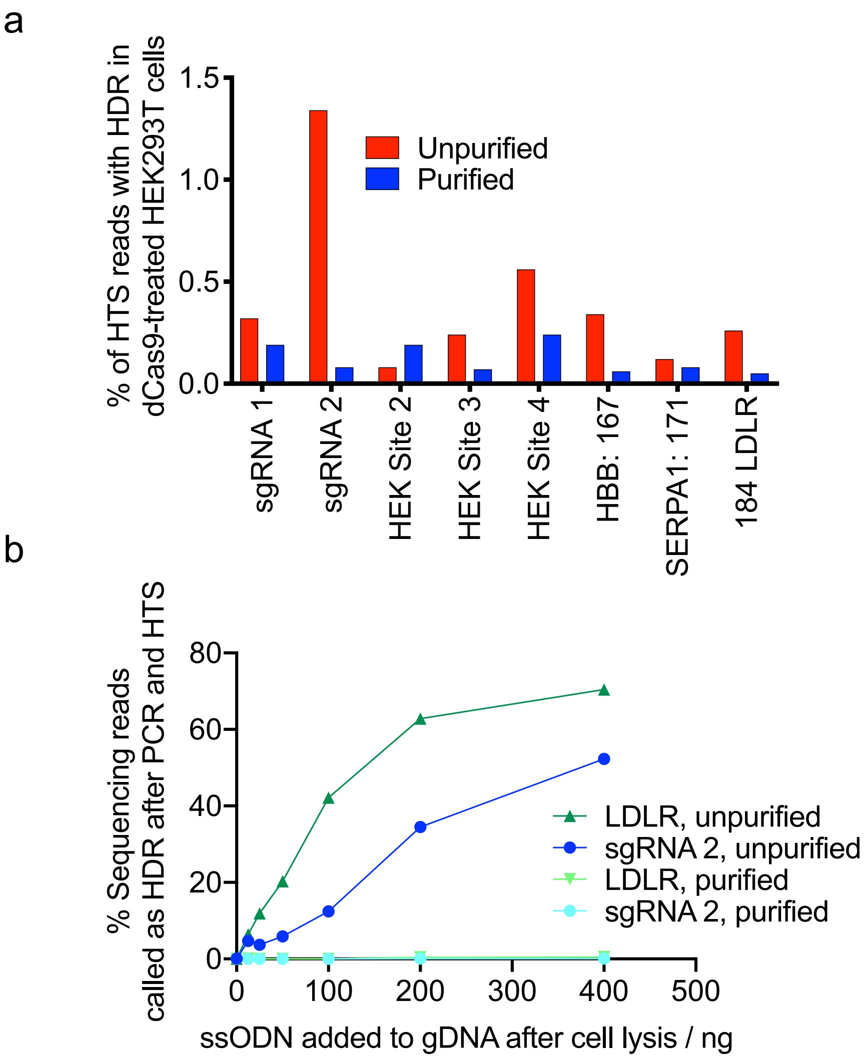

Supplementary Figure 7. Comparison of apparent HDR frequencies with and without magnetic bead-based purification of genomic DNA.
(a) HEK293T cells were lipofected with a plasmid encoding dCas9, a plasmid encoding the indicated sgRNA, and a 50 ng of a homologous 100-mer ssODN. Cells were lysed 4 days after treatment and crude cell lysate was saved before genomic DNA purification was performed with DNAdvance beads, as described in the Methods. The purified and unpurified genomic DNA samples were amplified by PCR and subjected to HTS, as described in the Methods. **(b)** Artifactual HDR frequencies recorded from addition of 100-mer ssODN to genomic DNA isolated from untreated HEK293T cells. The indicated ssODN was added to 600 ng genomic DNA and the resulting mixture subjected to PCR and HTS as described in the methods ("unpurified samples"). A sample of each ssODN and genomic DNA mixture was purified using Agincourt DNAdvance magnetic beads as described in the Methods ("purified samples") to assess the extent to which bead-based purification can separate genomic DNA from ssODN donor. Source data are provided as a Source Data file.

Example of hiPS cells treated with RDN(A89E)-P2A-GFP:z

single cell gates:

GFP + gate:

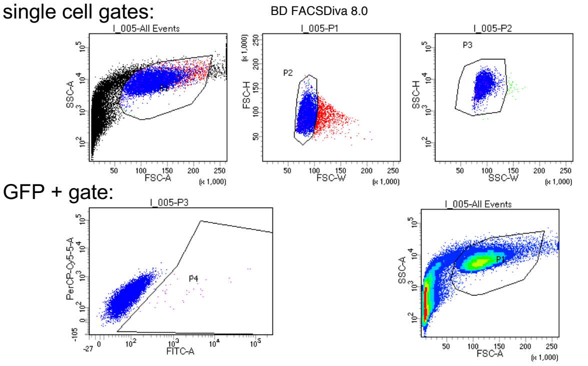

Tube: I_005

Population	#Events	%Parent	%Total
All Events	23,848	####	100.0
P1	11,063	46.4	46.4
P2	10,033	90.7	42.1
P3	10,000	99.7	41.9
P4	34	0.3	0.1

Example of negative control mock-nucleofected hiPS cells:

single cell gates:

GFP + gate:

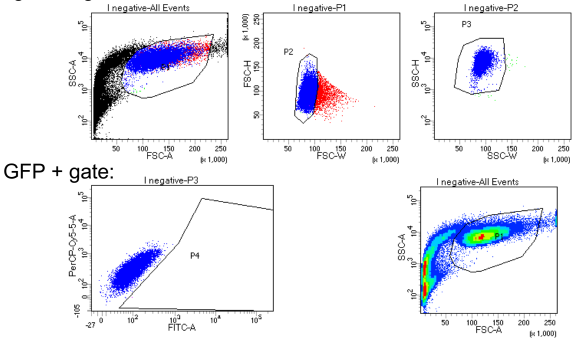

Tube: I negative

Population	#Events	%Parent	%Total
All Events	25,231	####	100.0
P1	11,934	47.3	47.3
P2	10,173	85.2	40.3
P3	10,145	99.7	40.2
P4	1	0.0	0.0

Example of hiPS cells treated with Cas9-P2A-GFP:

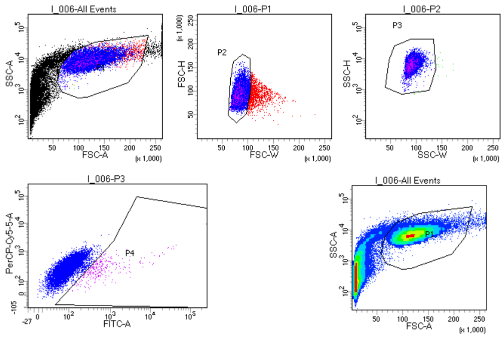

Tube: I_006

Population	#Events	%Parent	%Total
All Events	22,064	####	100.0
P1	11,341	51.4	51.4
P2	10,017	88.3	45.4
P3	10,000	99.8	45.3
P4	298	3.0	1.4

Supplementary Figure 8. Gating examples for flow sorting human iPSC cells (hiPSC).
See figures Figure 5i and 5j. Examples of flow sorting gates for single cells and for GFP+ cells are shown.

Supplementary Note 1. Indel formation and base editing arising from commonly used base editors at the genomic loci shown in Figure 1.

The Cas9 D10A nickase is a component of many DNA base editors, which are generally associated with low or undetectable indel rates[2,3]. We compared indel formation induced by D10A nickase to that associated with the recently reported expression-optimized base editors (ABEmax and BE4max)[4] and their predecessors (BE4[5] and ABE7.10[2]) (**Supplementary Figure 6a**). Base editors BE4max, ABE7.10, and BE4 are associated with lower indel rates than the D10A-nickase alone (average indel generation across 8 loci was 3.7±2.8% for the D10A nickase, 1.2±0.5% for BE4max, 1.2±0.7% for BE4 and 0.37±0.2% for ABE-7.10. ABEmax generated similar indel levels to the D10A-nickase alone, an average of 3.7±3.1%.

The basis for the elevated indel rates from optimizing ABE7.10 expression, which were not observed upon optimizing BE4 expression, is unclear, but may be attributed to increased levels of D10A nickase domain expression in ABEmax compared to ABE7.10, BE4max, or BE4[4]. These findings confirm that base editors generally induce lower indel rates than D10A nickase alone, and the elevated indel rates associated with ABEmax can be avoided by using ABE7.10[2].

sgRNA name	sgRNA + PAM sequence	Change made using ssODN template in HEK293T experiments	Change made using ssODN template in extended cell line experiments	Protein change induced by HDR
sgRNA 1	GAGCAAAGAGAATAGACTGT*AGG*	GAGCAAAGAGAATAGACTCT*ACA*		
sgRNA 2	GGATTGACCCAGGCCAGGGC*TGG*	GGATTGACCCAGGCGAGGGC*TGT*		
HEK2	GAACACAAAGCATAGACTGC*CGG*	GAACACAAAGCAGAGACTGC*CAC*		
HEK3	GGCCCAGACTGAGCACGTGA*TGG*	GGCCCAGACTGTGCACGTGA*TCC*	GGCCCAGACTGAGCAAGTGA*TGG*	
HEK4	GGCACTGCGGCTGGAGGTGG*GGG*	GGCACTGCGGCTGGAGTTGG*GAA*		
HBB	GTAACGGCAGACTTCTCCT*CAGG*	GTAACGGCAGACTTCTCCA*CAGG*	GTAACGGCAGACTTCTCCA*CAGG*	HBB: Glu6Val
SERPA1	[G]TGCTGACCATCGACGAGAAA*GGG*	TGCTGACCATCGACAAGAAA*GGG*		SERPINA1: Glu366Lys
LDLR	[G]CAGAGCACTGGAATTCGTCA*GGG*	CAGAGCACTGGAATTAGTCA*GGG*	CAGAGCACTGGAATTAGTCA*GGG*	LDLR: Glu240STOP

Supplementary Table 1. Single guide RNA (sgRNA) sequences and HDR products. PAM sequences are in italics. Nucleotides mutated through HDR are red. For sgRNAs SERPA1 and LDLR, a 5' G was included in the sgRNA expression cassette to enable efficient expression of the sgRNA from the U6 promoter. This 5' G is indicated as [G] in the sgRNA sequence column.
For sgRNAs 1, 2, HEK2, HEK3 and HEK4, a PAM mutation was incorporated into the ssODN template as well as the SNP indicated in the sgRNA protospacer sequence. HDR was quantified by the proportion of cells undergoing HDR that resulted in incorporation of the SNP in the protospacer, not in the PAM. The PAM mutation was not incorporated into the ssODN template used for extended cell line experiments at HEK site 3. Protein coding changes that would result from successful HDR have been listed in the final column.

8 core sites: used in Figs. 1,2,3 and Supplementary Figs. 2,3,5,7	
**sgRNA associated with oligo**	**ssODN Oligo Seqeuence**
sgRNA 1	ATTTTAAGCTGTAGTATTATGAAGGGAAATCTGGAGCAAAGAGAATAGACTCTACAGAAACCAGTTAAGAAATAGGACATGGAGGCTAGGTGCAGTGGCT
sgRNA 2	TTTCCTCTGCCATCACGTGCTCAGTCTGGGCCCCAAGGATTGACCCAGGACAGGGCTCGAGAAGCAGAAAAAAAGCATCAAGCCTACAAATGCATGCTTA
HEK2	TTTTCCAGCCCGCTGGCCCTGTAAAGGAAACTGGAACACAAAGCAGAGACTGCGACGCGGGCCAGCCTGAATAGCTGCAAACAAGTGCAGAATATCTGAT
HEK3	GCTTCTCCAGCCCTGGCCTGGGTCAATCCTTGGGGCCCAGACTGAGCAAGTGATTTCAGAGGAAAGGAAGCCCTGCTTCCTCCAGAGGGCGTCGCAGGAC
HEK4	GGATGACAGGCAGGGGCACCGCGGCGCCCCGGTGGCACTGCGGCTGGAGTTGGGAATTAAAGCGGAGACTCTGGTGCTGTGTGACTACAGTGGGGGCCCT
HBB	ACTTCATCCACGTTCACCTTGCCCCACAGGGCAGTAACGGCAGACTTCTCCACAGGAGTCAGATGCACCATGGTGTCTGTTTGAGGTTGCTAGTGAACAC
SERPA1	CATGGGTATGGCCTCTAAAAACATGGCCCCAGCAGCTTCAGTCCCTTTCTTGTCGATGGTCAGCACAGCCTTATGCACGGCCTGGAGGGGAGAGAAGCAG
LDLR	ATCAACACACTCTGTCCTGTTTTCCAGCTGTGGCCACCTGTCGCCCTGACTAATTCCAGTGCTCTGATGGAAACTGCATCCATGGCAGCCGGCAGTGTGA
**Figure 5**	**ssODN Oligo Seqeuence**
HBB	ACTTCATCCACGTTCACCTTGCCCCACAGGGCAGTAACGGCAGACTTCTCCACAGGAGTCAGATGCACCATGGTGTCTGTTTGAGGTTGCTAGTGAACAC
LDLR	ATCAACACACTCTGTCCTGTTTTCCAGCTGTGGCCACCTGTCGCCCTGACTAATTCCAGTGCTCTGATGGAAACTGCATCCATGGCAGCCGGCAGTGTGA
HEK3	GCTTCTCCAGCCCTGGCCTGGGTCAATCCTTGGGGCCCAGACTGAGCAAGTGATGGCAGAGGAAAGGAAGCCCTGCTTCCTCCAGAGGGCGTCGCAGGAC
**Figure 4a and Supplementary Figure 4**	
ssODNs in the "forward" sense - used in Fig 4a and Supplementary Fig. 4	
**Mutation in oligo**	**ssODN Oligo Seqeuence**
PAM mutation only	GCTTCTCCAGCCCTGGCCTGGGTCAATCCTTGGGGCCCAGACTGAGCACGTGATTTCAGAGGAAAGGAAGCCCTGCTTCCTCCAGAGGGCGTCGCAGGAC
SNP at 20 + PAM mutation	GCTTCTCCAGCCCTGGCCTGGGTCAATCCTTGGGGCCCAGACTGAGCACGTGTTTTCAGAGGAAAGGAAGCCCTGCTTCCTCCAGAGGGCGTCGCAGGAC
SNP at 18 + PAM mutation	GCTTCTCCAGCCCTGGCCTGGGTCAATCCTTGGGGCCCAGACTGAGCACGGGATTTCAGAGGAAAGGAAGCCCTGCTTCCTCCAGAGGGCGTCGCAGGAC
SNP at 16 + PAM mutation	GCTTCTCCAGCCCTGGCCTGGGTCAATCCTTGGGGCCCAGACTGAGCAAGTGATTTCAGAGGAAAGGAAGCCCTGCTTCCTCCAGAGGGCGTCGCAGGAC
SNP at 12 + PAM mutation	GCTTCTCCAGCCCTGGCCTGGGTCAATCCTTGGGGCCCAGACTGTGCACGTGATTTCAGAGGAAAGGAAGCCCTGCTTCCTCCAGAGGGCGTCGCAGGAC
SNP at 7 + PAM mutation	GCTTCTCCAGCCCTGGCCTGGGTCAATCCTTGGGGCCCACACTGAGCACGTGATTTCAGAGGAAAGGAAGCCCTGCTTCCTCCAGAGGGCGTCGCAGGAC
SNP at 25 + PAM mutation	GCTTCTCCAGCCCTGGCCTGGGTCAATCCTTGGGGCCCAGACTGAGCACGTGATTTCGGAGGAAAGGAAGCCCTGCTTCCTCCAGAGGGCGTCGCAGGAC
SNP at 28 + PAM mutation	GCTTCTCCAGCCCTGGCCTGGGTCAATCCTTGGGGCCCAGACTGAGCACGTGATTTCAGATGAAAGGAAGCCCTGCTTCCTCCAGAGGGCGTCGCAGGAC
SNP at 20 no PAM mutation	GCTTCTCCAGCCCTGGCCTGGGTCAATCCTTGGGGCCCAGACTGAGCACGTGTTGGCAGAGGAAAGGAAGCCCTGCTTCCTCCAGAGGGCGTCGCAGGAC
SNP at 18 no PAM mutation	GCTTCTCCAGCCCTGGCCTGGGTCAATCCTTGGGGCCCAGACTGAGCACGGGATGGCAGAGGAAAGGAAGCCCTGCTTCCTCCAGAGGGCGTCGCAGGAC
SNP at 16 no PAM mutation	GCTTCTCCAGCCCTGGCCTGGGTCAATCCTTGGGGCCCAGACTGAGCAAGTGATGGCAGAGGAAAGGAAGCCCTGCTTCCTCCAGAGGGCGTCGCAGGAC
SNP at 12 no PAM mutation	GCTTCTCCAGCCCTGGCCTGGGTCAATCCTTGGGGCCCAGACTGTGCACGTGATGGCAGAGGAAAGGAAGCCCTGCTTCCTCCAGAGGGCGTCGCAGGAC
SNP at 7 no PAM mutation	GCTTCTCCAGCCCTGGCCTGGGTCAATCCTTGGGGCCCACACTGAGCACGTGATGGCAGAGGAAAGGAAGCCCTGCTTCCTCCAGAGGGCGTCGCAGGAC
SNP at 25 no PAM mutation	GCTTCTCCAGCCCTGGCCTGGGTCAATCCTTGGGGCCCAGACTGAGCACGTGATGGCGGAGGAAAGGAAGCCCTGCTTCCTCCAGAGGGCGTCGCAGGAC
SNP at 28 no PAM mutation	GCTTCTCCAGCCCTGGCCTGGGTCAATCCTTGGGGCCCAGACTGAGCACGTGATGGCAGATGAAAGGAAGCCCTGCTTCCTCCAGAGGGCGTCGCAGGAC
**reverse ssODNs used in Supplementary Figure 4**	
PAM mutation only	GTCCTGCGACGCCCTCTGGAGGAAGCAGGGCTTCCTTTCCTCTGAAATCACGTGCTCAGTCTGGGCCCCAAGGATTGACCCAGGCCAGGGCTGGAGAAGC
SNP at 20 + PAM mutation	GTCCTGCGACGCCCTCTGGAGGAAGCAGGGCTTCCTTTCCTCTGAAAACACGTGCTCAGTCTGGGCCCCAAGGATTGACCCAGGCCAGGGCTGGAGAAGC
SNP at 18 + PAM mutation	GTCCTGCGACGCCCTCTGGAGGAAGCAGGGCTTCCTTTCCTCTGAAATCCCGTGCTCAGTCTGGGCCCCAAGGATTGACCCAGGCCAGGGCTGGAGAAGC
SNP at 16 + PAM mutation	GTCCTGCGACGCCCTCTGGAGGAAGCAGGGCTTCCTTTCCTCTGAAATCACTTGCTCAGTCTGGGCCCCAAGGATTGACCCAGGCCAGGGCTGGAGAAGC
SNP at 12 + PAM mutation	GTCCTGCGACGCCCTCTGGAGGAAGCAGGGCTTCCTTTCCTCTGAAATCACGTGCACAGTCTGGGCCCCAAGGATTGACCCAGGCCAGGGCTGGAGAAGC
SNP at 7 + PAM mutation	GTCCTGCGACGCCCTCTGGAGGAAGCAGGGCTTCCTTTCCTCTGAAATCACGTGCTCAGTGTGGGCCCCAAGGATTGACCCAGGCCAGGGCTGGAGAAGC
SNP at 25 + PAM mutation	GTCCTGCGACGCCCTCTGGAGGAAGCAGGGCTTCCTTTCCTCCGAAATCACGTGCTCAGTCTGGGCCCCAAGGATTGACCCAGGCCAGGGCTGGAGAAGC
SNP at 28 + PAM mutation	GTCCTGCGACGCCCTCTGGAGGAAGCAGGGCTTCCTTTCATCTGAAATCACGTGCTCAGTCTGGGCCCCAAGGATTGACCCAGGCCAGGGCTGGAGAAGC
SNP at 20 no PAM mutation	GTCCTGCGACGCCCTCTGGAGGAAGCAGGGCTTCCTTTCCTCTGCCAACACGTGCTCAGTCTGGGCCCCAAGGATTGACCCAGGCCAGGGCTGGAGAAGC
SNP at 18 no PAM mutation	GTCCTGCGACGCCCTCTGGAGGAAGCAGGGCTTCCTTTCCTCTGCCATCCCGTGCTCAGTCTGGGCCCCAAGGATTGACCCAGGCCAGGGCTGGAGAAGC
SNP at 16 no PAM mutation	GTCCTGCGACGCCCTCTGGAGGAAGCAGGGCTTCCTTTCCTCTGCCATCACTTGCTCAGTCTGGGCCCCAAGGATTGACCCAGGCCAGGGCTGGAGAAGC
SNP at 12 no PAM mutation	GTCCTGCGACGCCCTCTGGAGGAAGCAGGGCTTCCTTTCCTCTGCCATCACGTGCACAGTCTGGGCCCCAAGGATTGACCCAGGCCAGGGCTGGAGAAGC
SNP at 7 no PAM mutation	GTCCTGCGACGCCCTCTGGAGGAAGCAGGGCTTCCTTTCCTCTGCCATCACGTGCTCAGTGTGGGCCCCAAGGATTGACCCAGGCCAGGGCTGGAGAAGC
SNP at 25 no PAM mutation	GTCCTGCGACGCCCTCTGGAGGAAGCAGGGCTTCCTTTCCTCCGCCATCACGTGCTCAGTCTGGGCCCCAAGGATTGACCCAGGCCAGGGCTGGAGAAGC
SNP at 28 no PAM mutation	GTCCTGCGACGCCCTCTGGAGGAAGCAGGGCTTCCTTTCATCTGCCATCACGTGCTCAGTCTGGGCCCCAAGGATTGACCCAGGCCAGGGCTGGAGAAGC

Supplementary Table 2. Donor template sequences used for HDR.

Primers for amplification of genomic DNA	
LDLR forward	ACACTCTTTCCCTACACGACGCTCTTCCGATCTNNNNGCCCTGCTTCTTTTTCTCTGGT
LDLR reverse	TGGAGTTCAGACGTGTGCTCTTCCGATCTACCATTAACGCAGCCAACTTCA
HBB forward	ACACTCTTTCCCTACACGACGCTCTTCCGATCTNNNNGTCTTCTCTGTCTCCACATGCC
HBB reverse	TGGAGTTCAGACGTGTGCTCTTCCGATCTTAGGGTTGGCCAATCTACTCCC
HEK site 3 and sgRNA 2 forward	ACACTCTTTCCCTACACGACGCTCTTCCGATCTNNNNGGAAACGCCCATGCAATTAGTC
HEK site 3 and sgRNA 2 reverse	TGGAGTTCAGACGTGTGCTCTTCCGATCTCTTGTCAACCAGTATCCCGGTG
HEK site 2 forward	ACACTCTTTCCCTACACGACGCTCTTCCGATCTNNNNTGAATGGATTCCTTGGAAACAATG
HEK site 2 reverse	TGGAGTTCAGACGTGTGCTCTTCCGATCTCCAGCCCCATCTGTCAAACT
HEK site 4 forward	TGGAGTTCAGACGTGTGCTCTTCCGATCTTCCTTTCAACCCGAACGGAG
HEK site 4 reverse	ACACTCTTTCCCTACACGACGCTCTTCCGATCTNNNNGCTGGTCTTCTTTCCCCTCC
sgRNA 1 forward	ACACTCTTTCCCTACACGACGCTCTTCCGATCTNNNNGAGTTACTGCTCAGACATGTAA
sgRNA 1 reverse	TGGAGTTCAGACGTGTGCTCTTCCGATCTGACCTCGTGATCCACCTGCC
SERPA1 forward	ACACTCTTTCCCTACACGACGCTCTTCCGATCTNNNNTTTGTTGAACTTGACCTCGGGG
SERPA1 reverse	TGGAGTTCAGACGTGTGCTCTTCCGATCTCATCAGCCAAAGCCTTGAGGAG

Supplementary Table 3. DNA primers used for amplification of genomic DNA prior to HTS.

Relevant to Figure 2	sgRNA 1	sgRNA 2	HEK Site 2	HEK Site 3	HEK Site 4:	HBB	SERPA1	LDLR
Cas9 vs. i53	0.027	0.002	0.494	0.083	0.812	0.017	0.394	0.020
Cas9 vs. hRad51	0.000	0.002	0.055	0.022	0.038	0.051	0.000	0.254
Cas9 vs. hRadK133R	0.022	0.951	0.007	0.206	0.050	0.855	0.031	0.343
Cas9 vs. hRad52	0.251	0.029	0.017	0.278	0.292	0.041	0.516	0.549
D10A VS. hRad52	0.946	0.892	0.534	0.643	0.034	0.818	0.026	0.276
D10A vs. hRad-K133R	0.142	0.004	0.003	0.100	0.020	0.003	0.011	0.052
D10A vs. i53	0.028	0.274	0.136	0.204	0.683	0.004	0.572	0.843
D10A vs. hRad51	0.013	0.146	0.084	0.111	0.110	0.001	0.042	0.906
**Relevant to Figure 3**								
Cas9 vs. hRad51(K133R)-Cas9	0.004	0.806	0.008	0.486	0.553	0.024	0.005	0.001
D10A vs. hRad51(K133R)-D10A	0.242	0.007	0.009	0.039	0.021	0.014	0.003	0.028
H840A vs. hRad51(K133R)-H840A	0.002	0.057	0.025	0.859	0.052	0.008	0.374	0.374
hRad51(K133R)-D10A vs. hRad51-D10A	0.836	0.443	0.282	0.929	0.734	0.862	0.251	0.787
hRad51(F86E)-D10A vs. hRad51-D10A	0.001	0.287	0.019	0.514	0.007	0.044	0.015	0.001
hRad51(A89E)-D10A vs. hRad51-D10A	0.000	0.066	0.008	0.322	0.035	0.049	0.015	0.001
hRad51(A190L,A192L)-D10A vs. hRad51-D10A	0.000	0.115	0.060	0.198	0.042	0.327	0.442	0.006
hRad51(SA208,209ED)-D10A vs. hRad51-D10A	0.001	0.847	0.331	0.414	0.119	0.426	0.000	0.022

Supplementary Table 4. P-values for comparisons between conditions for absolute HDR frequencies in HEK293T cells.

Student's two-tailed, two-sample equal variance t test was performed in Excel. Values have been colored according to p-value; green = $p>0.05$, yellow = $0.01<p<0.05$ and red = $p<0.01$.

	sgRNA 1	sgRNA 2	HEK Site 2	HEK Site 3	HEK Site 4:	HBB	SERPA1
**Relevant to Figure 2**							
Cas9 vs. i53	0.01	0.03	0.09	0.12	0.64	0.00	0.07
Cas9 vs. hRad51	0.00	0.00	0.01	0.05	0.11	0.18	0.01
Cas9 vs. hRadK133R	0.03	0.11	0.81	0.02	0.27	0.08	0.02
Cas9 vs. hRad52	0.24	0.12	0.05	0.75	0.30	0.59	0.06
D10A VS. hRad52	0.00	0.14	0.17	0.04	0.04	0.02	#DIV/0!
D10A vs. hRad-K133R	0.54	0.38	0.03	0.58	0.81	0.28	#DIV/0!
D10A vs. i53	0.21	0.33	0.20	0.57	0.30	0.20	#DIV/0!
D10A vs. hRad51	0.01	0.12	0.02	0.07	0.12	0.18	#DIV/0!
**Relevant to Figure 3**							
Cas9 vs. hRad51(K133R)-Cas9	0.00	0.22	0.05	0.20	0.74	0.03	0.11
D10A vs. hRad51(K133R)-D10A	0.53	0.85	0.01	0.83	0.42	0.13	#DIV/0!
H840A vs. hRad51(K133R)-H840A	0.02	0.11	0.70	#DIV/0!	0.37	#DIV/0!	#DIV/0!
hRad51(K133R)-D10A vs. hRad51-D10A	0.64	0.74	0.06	0.19	0.90	0.14	#DIV/0!
hRad51(F86E)-D10A vs. hRad51-D10A	0.33	0.25	0.02	0.01	0.16	0.14	0.10
hRad51(A89E)-D10A vs. hRad51-D10A	0.23	0.20	0.01	0.01	0.07	0.33	0.13
hRad51(A190L,A192L)-D10A vs. hRad51-D10A	0.23	0.58	0.80	0.26	0.37	0.05	0.24
hRad51(SA208,209ED)-D10A vs. hRad51-D10A	0.13	0.05	0.00	0.07	0.99	0.00	0.07

Supplementary Table 5. P-values for comparisons between conditions for HDR:indel ratios in HEK293T cells.

Student's two-tailed, two-sample equal variance t test was performed in Excel. Values have been colored according to p-value; green = p>0.05, yellow = 0.01<p<0.05 and red = p<0.01. #Div0 error occurred when HDR:indel ratio could not be calculated due to values of HDR lower than 1% to avoid inflating HDR:indel ratios for low values of HDR (see Methods).

Plasmid Name	Addgene Number
hRad51(S208E-A209D)–Cas9(D10A)	125561
hRad51(A190L-A192L)–Cas9(D10A)	125562
hRad51(A89E)–Cas9(D10A)	125563
hRad51(F86E)–Cas9(D10A)	125564
hRad51(G151D)–Cas9(D10A)	125565
hRad51–Cas9(D10A)	125567
hRad51(K133R)–Cas9(D10A)	125568
hRad51(K133R)	125571
hRad51	125570

Supplementary Table 6. Accession numbers for deposited plasmids with Addgene.

Supplementary References

1 Shen, M. W. *et al.* Predictable and precise template-free CRISPR editing of pathogenic variants. *Nature* **563**, 646-651, doi:10.1038/s41586-018-0686-x (2018).

2 Gaudelli, N. M. *et al.* Programmable base editing of A*T to G*C in genomic DNA without DNA cleavage. *Nature* **551**, 464-471, doi:10.1038/nature24644 (2017).

3 Komor, A. C., Kim, Y. B., Packer, M. S., Zuris, J. A. & Liu, D. R. Programmable editing of a target base in genomic DNA without double-stranded DNA cleavage. *Nature* **533**, 420-424, doi:10.1038/nature17946 (2016).

4 Koblan, L. W. *et al.* Improving cytidine and adenine base editors by expression optimization and ancestral reconstruction. *Nat Biotechnol* **36**, 843-846, doi:10.1038/nbt.4172 (2018).

5 Komor, A. C. *et al.* Improved base excision repair inhibition and bacteriophage Mu Gam protein yields C:G-to-T:A base editors with higher efficiency and product purity. *Sci Adv* **3**, eaao4774, doi:10.1126/sciadv.aao4774 (2017).
