# Supplementary material for: Development of hRad51–Cas9 nickase fusions that mediate HDR without double-stranded breaks
Source: Nat Commun. 2019 May 17;10:2212. doi: 10.1038/s41467-019-09983-4 (PMC6525190; doi:10.1038/s41467-019-09983-4)
Supplement: Supplementary file 2 — Reporting Summary [file 41467_2019_9983_MOESM2_ESM.pdf]

nature research

Corresponding author(s): David R. Liu

Last updated by author(s): April 1, 2019

Reporting Summary

Nature Research wishes to improve the reproducibility of the work that we publish. This form provides structure for consistency and transparency in reporting. For further information on Nature Research policies, see Authors & Referees and the Editorial Policy Checklist.

Statistics

For all statistical analyses, confirm that the following items are present in the figure legend, table legend, main text, or Methods section.

n/a	Confirmed	
☐	☒	The exact sample size (n) for each experimental group/condition, given as a discrete number and unit of measurement
☒	☐	A statement on whether measurements were taken from distinct samples or whether the same sample was measured repeatedly
☐	☒	The statistical test(s) used AND whether they are one- or two-sided<br>*Only common tests should be described solely by name; describe more complex techniques in the Methods section.*
☒	☐	A description of all covariates tested
☒	☐	A description of any assumptions or corrections, such as tests of normality and adjustment for multiple comparisons
☒	☐	A full description of the statistical parameters including central tendency (e.g. means) or other basic estimates (e.g. regression coefficient) AND variation (e.g. standard deviation) or associated estimates of uncertainty (e.g. confidence intervals)
☒	☐	For null hypothesis testing, the test statistic (e.g. F, t, r) with confidence intervals, effect sizes, degrees of freedom and P value noted<br>*Give P values as exact values whenever suitable.*
☒	☐	For Bayesian analysis, information on the choice of priors and Markov chain Monte Carlo settings
☒	☐	For hierarchical and complex designs, identification of the appropriate level for tests and full reporting of outcomes
☒	☐	Estimates of effect sizes (e.g. Cohen's d, Pearson's r), indicating how they were calculated

Our web collection on statistics for biologists contains articles on many of the points above.

Software and code

Policy information about availability of computer code

Data collection	For data collection of high throughput sequencing (HTS) reads, a demultiplexing of barcoded reads was performed by the MiSeq Reporter Software (Illumina)
Data analysis	For analysis of sequencing reads obtained from Illumina Miseq amplicon sequencing, Crispresso-2 was used for alignment and indel quantification. This software has been published here: https://www.biorxiv.org/node/118932.abstract, and has been accepted for publication in Nature Biotechnology (currently in press).

For manuscripts utilizing custom algorithms or software that are central to the research but not yet described in published literature, software must be made available to editors/reviewers. We strongly encourage code deposition in a community repository (e.g. GitHub). See the Nature Research guidelines for submitting code & software for further information.

Data

Policy information about availability of data

All manuscripts must include a data availability statement. This statement should provide the following information, where applicable:

- Accession codes, unique identifiers, or web links for publicly available datasets
- A list of figures that have associated raw data
- A description of any restrictions on data availability

Plasmids encoding the constructs used in this study will be available on Addgene after publication. High-throughput DNA sequencing data has been deposited in the NCBI Sequence Read Archive with BioProject accession number PRJNA515942 (SRP180368)

Field-specific reporting

Please select the one below that is the best fit for your research. If you are not sure, read the appropriate sections before making your selection.

☒ Life sciences ☐ Behavioural & social sciences ☐ Ecological, evolutionary & environmental sciences

For a reference copy of the document with all sections, see nature.com/documents/nr-reporting-summary-flat.pdf

Life sciences study design

All studies must disclose on these points even when the disclosure is negative.

Sample size	No sample size calculation was performed
Data exclusions	No data were excluded
Replication	Replicates were performed independently as indicated in the figure legends. Wherever possible, different splits of cells and different preparations of plasmid DNA were used.
Randomization	This is not relevant to the submitted work.
Blinding	This is not relevant to the submitted work.

Reporting for specific materials, systems and methods

We require information from authors about some types of materials, experimental systems and methods used in many studies. Here, indicate whether each material, system or method listed is relevant to your study. If you are not sure if a list item applies to your research, read the appropriate section before selecting a response.

Materials & experimental systems

n/a	Involved in the study	
☒	☐	Antibodies
☐	☒	Eukaryotic cell lines
☒	☐	Palaeontology
☒	☐	Animals and other organisms
☒	☐	Human research participants
☒	☐	Clinical data

Methods

n/a	Involved in the study	
☒	☐	ChIP-seq
☐	☒	Flow cytometry
☒	☐	MRI-based neuroimaging

Eukaryotic cell lines

Policy information about cell lines

Cell line source(s)	HEK293T, HeLa, U2OS and K562 cells were obtained from ATCC. Human iPSCs were obtained from Thermo Fisher (A18945) as described in the Methods.
Authentication	Cells were authenticated by the vendor.
Mycoplasma contamination	Cells were tested by the vendor and at regular intervals (once every six months) for mycoplasma in our lab. They tested negative.
Commonly misidentified lines (See ICLAC register)	N/A

Flow Cytometry

Plots

Confirm that:

☒ The axis labels state the marker and fluorochrome used (e.g. CD4-FITC).

☒ The axis scales are clearly visible. Include numbers along axes only for bottom left plot of group (a 'group' is an analysis of identical markers).

☒ All plots are contour plots with outliers or pseudocolor plots.

☒ A numerical value for number of cells or percentage (with statistics) is provided.

Methodology

Sample preparation	Cells were grown in tissue culture, nucleofected as described in methods. After 3 to 4 days, cells were dissociated with Accutase and filtered to remove debris before sorting.
Instrument	FACSAria
Software	BD FACS DIVA software was used for analysis.
Cell population abundance	The abundance of cells in the post-sort fraction was dependent upon cell type and sorting condition. Nucleofection with ssODN, sgRNA, and Cas9-P2A-GFP or hRad51-P2A-GFP GFP yield 0% to 3% GFP positive cells.
Gating strategy	Negative control (unstained) and GFP-positive cells were used to establish gates for iPSCs. Gates were drawn to collect cells expressing GFP. See the provided examples for gates used.

☒ Tick this box to confirm that a figure exemplifying the gating strategy is provided in the Supplementary Information.
